# Meta-Analysis of Randomized Controlled Trials on the Efficacy and Safety of Donepezil, Galantamine, Rivastigmine, and Memantine for the Treatment of Alzheimer’s Disease

**DOI:** 10.3389/fnins.2019.00472

**Published:** 2019-05-15

**Authors:** Dan-Dan Li, Ya-Hong Zhang, Wei Zhang, Pu Zhao

**Affiliations:** ^1^College of Life and Health Sciences, Northeastern University, Shenyang, China; ^2^Department of Hepatobiliary Surgery, General Hospital of Northern Theater Command, Shenyang, China

**Keywords:** Alzheimer’s disease, donepezil, galantamine, memantine, rivastigmine, meta-analysis

## Abstract

To study the impact of donepezil, rivastigmine, galantamine, and memantine on cognitive, functional, behavioral, global changes and adverse effects in patients with mild, moderate and severe Alzheimer’s disease (AD), we screened the literature published before September 2017 in the Pubmed, Embase, Cochrane library and Web of Science Electronic databases according to the inclusion criteria. Thirty-six studies were finally determined from 1560 preliminary screened articles. The AD Assessment Scale-cognitive Subscale (ADAS-cog), AD Cooperative Study-Activities of Daily Living (ADCS-ADL), Neuropsychiatric Inventory (NPI), and Clinician’s Interview-Based Impression of Change Plus Caregiver Input scale (CIBIC+) were used as valid endpoints. Of the 36 trials included, meta-analyses of these placebo-control trials showed that there were significant differences between the donepezil, rivastigmine and placebo groups using ADAS-cog, ADCS-ADL, and CIBIC+. Meta-analyses of these placebo-controlled trials showed that there were significant differences between the galantamine and placebo groups using ADAS-cog, ADCS-ADL, NPI, and CIBIC+. These observations suggest that memantine is beneficial for stabilizing or slowing the decline in ADAS-cog and ADCS-ADL_19_ changes in AD patients. However, there was no significant effect according to the ADCS-ADL_23_, NPI, and CIBIC+ tests, which indicated that memantine treatment has no significant effect on these cognitive aspects of AD patients. Different effects of donepezil, rivastigmine, galantamine, or memantine on AD were found in this study. According to the results, we conclude that galantamine is effective in treating all aspects of AD and is the first choice for the treatment of AD. However, due to limited data, we should consider additional data to obtain more stable results.

## Introduction

The relationship between cognitive dysfunction or impairment and Alzheimer’s disease AD has been reported in the literature (Stern et al., 1990; Chen et al., 1998; Perry and Hodges, 2000; Caro et al., 2002; Pereira et al., 2008). Meanwhile, cholesterol esterase inhibitors (ChEs) and memantine (Supplementary Table 1), which is a non-competitive *N*-methyl-D-aspartate (NMDA) receptor antagonist, can normalize dysfunctional glutamatergic neurotransmission (Parsons et al., 2013), which has shown effective efficacy in the treatment of AD. Since the intrinsic mechanism of acetylcholinesterase inhibitors (AChE-Is) requires a sufficient amount of residual endogenous acetylcholine, which is available, the therapeutic efficacy is expected to decrease with the severity of dementia. Cholinesterase inhibitors, which can reduce acetylcholine breakdown in the brain, are widely considered as a treatment options for AD (Doody et al., 2001). Therefore, greater atrophy in the brain regions that are responsible for the cholinergic pathway was found in those patients who had no response to donepezil (Bottini et al., 2012). It has been reported that donepezil is a type of AChE-I that improves cerebral blood flow (CBF), as well as its primary effect on memory function (Kogure et al., 2017). Galantamine is a newly available cholinergic drug that counteracts AD by specifically and reversibly inhibiting acetylcholinesterase (AChE) and altering the nicotinic cholinergic receptors, thereby subsequently reducing central cholinergic neurotransmission (Tariot, 2001). The deterioration of cognitive function in patients with AD appears to be mediated by the use of cholinergic drugs such as rivastigmine (Birks, 2006). Rivastigmine is a novel brain-selective inhibitor of “pseudo-irreversible𠇍 AChE, whose metabolism is almost completely independent of the cytochrome P450 system (Sramek et al., 1996). Memantine protects neurons against the overstimulation of NMDA receptors, which occurs in AD and thus causes glutamate- and calcium-mediated neurotoxicity (Jiang and Jiang, 2015). Evidence of the efficacy of memantine has been shown primarily in patients with moderate or severe AD (Areosa et al., 2005). The etiology and pathogenesis of AD are not well understood, but central cholinergic neurons are found to be impaired in AD patients with low choline intake and reduced choline synthesis (Inestrosa et al., 2005). The central cholinergic system plays a key role in regulating learning, memory and attention. AChE-Is is a major drug in the clinical management of AD (Tan et al., 2014), which can improve cognitive function by prolonging the duration of action of acetylcholine (Ach) in the CNS to improve cholinergic function and slow down memory loss. AchE-Is such as donepezil and galantamine show a significant effect on mild-to-moderate AD (Winblad et al., 2001; Raskind et al., 2004). Donepezil, rivastigmine and galantamine, which belong to the group of ChE inhibitors (ChE-Is), are capable of cognitive, functional and behavioral improvement; however, none of them has been shown to be effective in the progression of AD (Zemek et al., 2014). Currently, AchE-Is, including donepezil, rivastigmine, and galantamine, are standard treatments for slowing disease progression (Li et al., 2015). In addition, high dropout rates and adverse-effect-induced dropouts were observed in randomized clinical trials of these drugs. Therefore, the safety of ChE-Is and memantine has been proposed.

We conducted a systematic review and meta-analysis of donepezil, galantamine, rivastigmine and memantine in AD to elucidate the efficacy and safety of these drugs. We sought to elaborate on previous reviews and include a broad range of outcome measures to determine the extent to which these drugs have varying degrees of effects on cognitive, behavioral and functional impairment in AD patients at different stages of severity.

## Materials and Methods

### Search Strategy

PubMed (from 1966 to September 2017), EMBASE (from 1980 to September 2017), Web of science (from 1986 to September 2017) and the Cochrane Library (September 2017) were searched. The following search terms were used: “Alzheimer Disease” or “AD,” “Donepezil,” “Galantamine,” “Memantine,” “Rivastigmine,” or combination of these words.

### Selection Criteria

The inclusion criteria for the meta-analyses were as follows: (1) full-text publications written in English; (2) double-blind, parallel-group, placebo-controlled, with random assignment to donepezil, rivastigmine, galantamine, or memantine; (2) inclusion of patients with or probably with AD diagnosis, according to the fourth edition of the Mental Disorders Diagnostics and Statistics Manual (DSM-IV) and the National Association of Nervous and Communicative Disorders and Stroke/Alzheimer’s Disease Institute of Standards (NINCDS-ADRDA) (McKhann et al., 1984; American Psychiatric Association, 2000); (3) includes treatment duration for at least 52 weeks and at least one measure that reflects changes in cognitive, functional, behavioral or global assessment of change, as well as the number of adverse events (AEs) that led to dropout, and AE changes; (4) drug dosage and dosage form specifications. Studies with fatal defects in research design or data analysis were excluded, and trials with no readily available data were also excluded.

Studies were excluded for the following reasons: (1) not randomized controlled trials, such as case reports, reviews, and meta-analysis; (2) family-based studies; (3) lack of original data, such as meeting abstracts, and case reports/series; (4) non-human studies; and (5) publication in a language other than English.

### Search Strategy and Selection Criteria

Data abstraction was performed as a collaboration between two researchers using standard data extraction by discussing with other team members or by asking for the exact data from the original investigators. For missing data, we sought missing information and essential clarification from the authors. For measure variables, there is an approximate or direct algebraic relationship with standard deviation (SD), which we obtained from the standard error, confidence interval, *t*-value, or *p*-value related to the differences between the two sets of means.

We obtained the following baseline variables from each study: sample size, age, sex, race, design, dosing, blinding, duration of the trial, baseline cognitive score (Mini-Mental State Examination, MMSE), random numbers, secondary outcomes, and AEs that led to dropouts during the double-blind trials.

### Types of Outcome Measures

The measurement scales used in the tests varied. Therefore, we recorded measurement scales based on the general areas being assessed, namely, cognition, function, behavior and global assessment of change. Next, we attempted to determine a single measurement scale, which is the most commonly used key outcome measure in each area. We engaged the ADAS-cog, Alzheimer’s Disease Cooperative Research-Activities of Daily Living (ADCS-ADL), NPI and Clinician’s Interview-Based Impression of Change Plus Caregiver Input scale (CIBIC+) as primary measurements to assess the effects of different drugs on the cognition, function, behavior, and global assessment of change (Supplementary Table 2). The Alzheimer’s Disease Cooperative Study Activities of Daily Living 19- or 23-item scale (ADCS-ADL 19/23), which is based on interviews with caregivers or others close to the patients, was used as a scale to assess how the patients cope with daily activities. The 19-item subset was used for patients with moderate to severe AD, and the 23-item subset was used for patients with mild to moderate AD. In addition, to assess the efficacy and safety of these drugs, we recorded discontinuations of the trials due to AEs and dropouts due to adverse effects and other reasons.

### Statistical Analysis

Meta-analysis was performed using the Review Manager 5.3 software. For continuous data collected using the same measurement scale (e.g., cognition and behavior), we calculated the mean difference (MD) or standardized mean differences (SMD) with 95% confidence intervals (CIs) for changes from the baseline or final values. For dichotomous clinical outcomes, dropouts, and AEs, we performed odds ratios (ORs), absolute risk differences, 95% CI and *p* values to assess the efficacy and safety of the studied drugs. Heterogeneity was assessed using the Cochran Q-statistic and *I*^2^ tests (Doody et al., 2001). *I*^2^ approximates the ratio of the total variance in effect estimates due to heterogeneity rather than sampling error. Heterogeneity of the index is considered when *p* < 0.10 and *I*^2^> 50%. The latent retrieval bias was evaluated by the MD or SMD funnel plot in the main results of each test.

## Results

### Literature Search Findings

**FIGURE 1:**
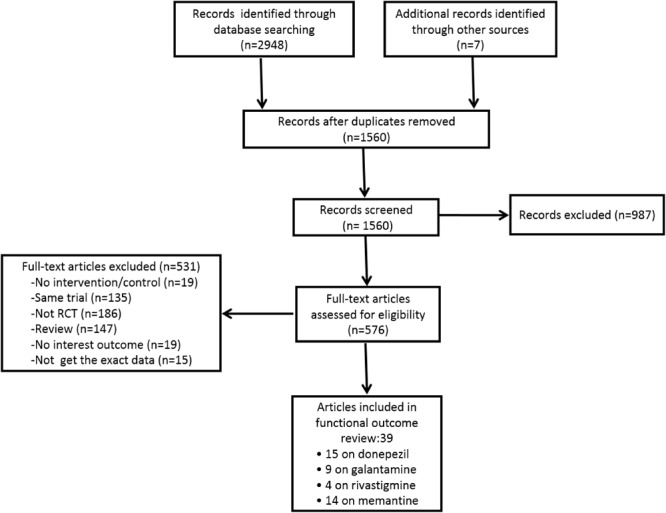
Flowchart describing the approach used to identify all eligible studies of meta-analysis.

The search strategy yielded 1,560 citations in PubMed, EMBASE, Web of Science, and the Cochrane Library of Systematic Reviews. Figure 1 shows the results of the literature search and study selection. A total of 573 potentially relevant articles were identified in the original search, but only 36 were ultimately selected for meta-analysis. Of these, fifteen donepezil trials (Feldman et al., 2001; Tariot et al., 2001; Krishnan et al., 2003; Tune et al., 2003; Seltzer et al., 2004; Johannsen et al., 2006; Winblad et al., 2006; Black et al., 2007; Homma et al., 2008; Frölich et al., 2011; Maher-Edwards et al., 2011; Haig et al., 2014; Gault et al., 2015, 2016; Jia et al., 2017), seven galantamine trials (Raskind et al., 2000; Tariot et al., 2000; Wilcock et al., 2000; Rockwood et al., 2001; Wilkinson and Murray, 2001; Brodaty et al., 2005; Burns et al., 2009), four rivastigmine trials (Forette et al., 1999; Rosler et al., 1999; Feldman and Lane, 2007; Winblad et al., 2007), and ten memantine trials (Reisberg et al., 2003; Tariot et al., 2004; Peskind et al., 2006; van Dyck et al., 2007; Bakchine and Loft, 2008; Porsteinsson et al., 2008; Fox et al., 2012; Grossberg et al., 2013; Herrmann et al., 2013; Wang et al., 2013) were included in the review. The design and population characteristics of the ChE-Is and memantine tests are shown in Table 1 and Supplementary Tables 3 – 5.

**Table 1:** Baseline characteristics of the studies included in the meta-analysis, by study donepezil drug.

Study	Country	Dose (number of patient)	Gender (%men)	Age years (SD)	Disease Severity	Type of Drug dosing	Duration (weeks)	Baseline MMSE (SD)	Outcomes				Dropout rate (%)	Number of adverse events caused dropout	Number of any adverse events
									Cognition	Function	Behavior	Global			
Feldman et al., 2001	Canada	Placebo (146)	39	74.0 ± 11	Moderate to severe	Flexible	24	11.9 ± 4.11	–	–	NPI	–	13.7	9	117
		10 mg daily (144)	38.9	73.3 ± 10				11.7 ± 4.2					16.0	12	120
Tariot et al., 2001	United States	Placebo (105)	18	85.9 ± 9.25	Mild to moderate	Fixed	24	14.4 ± 5.8	–	–	NPI	–	–	–	102
		10 mg daily (103)	17	85.4 ± 8.5				14.4 ± 5.4					–	–	99
Krishnan et al., 2003	United States	Placebo (33)	30	72.4 ± 10.1	Mild to moderate	Flexible	24	19.0 ± 4.6	ADAS-cog	–	–	–	30.3	1	–
		10 mg daily (34)	26	74.4 ± 7.0				19.5 ± 4.8					17.6	0	–
Tune et al., 2003	United States	Placebo (14)	28.6	72.2 ± 9.57	Mild to moderate	Fixed	24	21.4 ± 4.1	ADAS-cog	–	NPI	–	–	–	–
		10 mg daily (14)	21.4	73.7 ± 5.25				20.8 ± 3.7					–	–	–
Seltzer et al., 2004	United States	Placebo (57)	50	73.3 ± 8.8	Mild to moderate	Fixed	24	24.3 ± 1.3	ADAS-cog	–	–	–	19.3	5	37
		10 mg daily (96)	40	73.3 ± 9.6				24.1 ± 1.7					27.1	15	67
Johannsen et al., 2006	Denmark	Placebo (103)	36.9	71.4 ± 9.3	Mild to moderate	Fixed	12	18.5 ± 4.8	ADAS-cog	–	NPI	–	19.4	–	33
		10 mg daily (99)	40.4	74.1 ± 7.6				18.8 ± 4.8					11.1	–	27
Winblad et al., 2006	Sweden	Placebo (120)	26	85.3 ± 5.9	Severe	Fixed	24	5.8 ± 3.1	–	ADCS-ADL	NPI	–	17.5	8	–
		10 mg daily (128)	21	84.5 ± 6.0				6.3 ± 3.0					25.8	20	–
Black et al., 2007	Canada	Placebo (167)	27.3	78.0 ± 8.04	Severe	Fixed	24	7.4 ± 3.57	–	ADCS-ADL	NPI	CIBIC+	24.7	18	117
		10 mg daily (176)	32.3	78.0 ± 8.20				7.5 ± 3.25					34.9	34	140
Homma et al., 2008	Japan	Placebo (102)	17.6	79.7 ± 7.5	Severe	Flexible	24	8.0 ± 3.3	–	ADCS-ADL	–	CIBIC+	20.0	11	–
a		5 mg daily (96)	20.8	78.0 ± 8.90				7.9 ± 3.3					12.9	8	–
b		10 mg daily (92)	20.7	76.9 ± 7.9				7.4 ± 3.4					12.9	13	–
Maher-Edwards et al., 2011	United Kingdom	Placebo (61)	30	71.6 ± 6.72	Mild to moderate	Fixed	24	18.3 ± 3.36	ADAS-cog	–		CIBIC+	23.8	4	18
		10 mg daily (67)	37	71.1 ± 8.39				19.2 ± 3.20					14.9	1	26
Frölich et al., 2011	Germany	Placebo (163)	44.8	73.5 ± 6.42	Mild to moderate	Flexible	12	20.7 ± 3.96	ADAS-cog	–	–	–	11.6	–	60
c		5/10 mg daily (158)	34.2	73.9 ± 6.48				20.6 ± 3.90					13.7	–	60
Haig et al., 2014	United States	Placebo (63)	38.1	70.3 ± 7.84	Mild to moderate	Fixed	12	18.2 ± 3.9	ADAS-Cog	ADCS-ADL	NPI		39.7	3	25
		10 mg daily (60)	40.0	70.5 ± 8.31				18.1 ± 4.1					31.7	4	30
Gault et al., 2015	United States	Placebo (68)	38.2	73.6 ± 8.23	Mild to moderate	Fixed	12	19.7 ± 3.95	ADAS-cog	ADCS-ADL	NPI	–	4.4	2	26
		10 mg daily (68)	54.4	73.9 ± 7.92				19.6 ± 3.82					8.8	5	29
Gault et al., 2016	United States	Placebo (104)	37.5	73.2 ± 7.39	Mild to moderate	Fixed	24	19.1 ± 4.00	ADAS-cog	ADCS-ADL	NPI	–	14.4	3	56
		10 mg daily (76)	46.7	75.1 ± 7.75				18.4 ± 4.42					19.7	7	47
Jia et al., 2017	China	Placebo (156)	37.8	70.0 ± 9.57	Severe	Flexible	24	7.0 ± 3.40	–	–	–	CIBIC+	19.2	10	–
		10 mg daily (157)	32.5	71.6 ± 8.56				7.6 ± 3.36					18.4	14	–

*ADAS-cog, Alzheimer’s Disease (AD) Assessment Scale, cognitive subscale (possible range 0–70); ADCS-ADL, AD Cooperative Study Activities of Daily Living Inventory; ADCS-ADLsev, Alzheimer’s Disease Cooperative Study Activities of Daily Living Inventory modified for severe dementia; CIBIC+, Clinicians’ Interview-Based Impression of Change with Caregiver’s Input (possible range 1–7); MMSE Mini-Mental State Examination, NPI Neuropsychiatric Inventory, a, b, c different doses of drug, – Not reported.*

### Effects of Interventions

#### Cognitive Function

##### Donepezil

**FIGURE 2:**
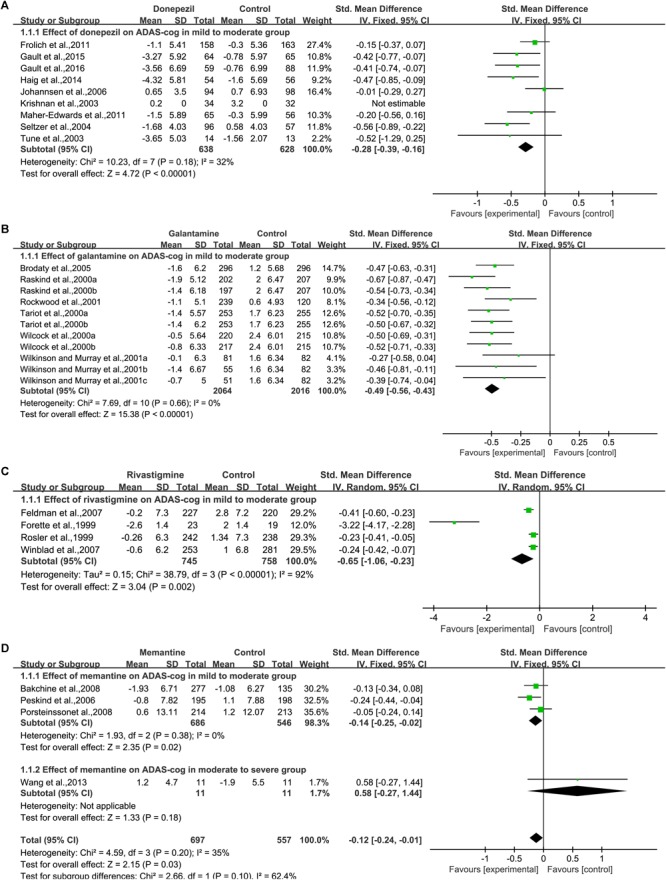
Cognitive outcomes on the ADAS-cog subscale (change from baseline) in AD patients in trials of cholinesterase inhibitors (**A**, Donepezil; **B**, Galantamine; **C**, Rivastigmine) and memantine **(D)**, according to drug and dose.

Nine studies (Tariot et al., 2001; Krishnan et al., 2003; Seltzer et al., 2004; Johannsen et al., 2006; Frölich et al., 2011; Maher-Edwards et al., 2011; Haig et al., 2014; Gault et al., 2015, 2016) assessed changes in cognition by using the ADAS-cog (Figure 2A). Significant cognition changes were found via meta-analysis in the available data, when compared with placebo. The SMD in the changes between the donepezil and placebo groups varied [SMD = –0.28, 95% CI (–0.39, –0.16); *p* < 0.00001]. The heterogeneity among most pooled studies was low (*p* = 0.18, *I*^2^= 32%). The funnel plots (data not shown) did not show symmetric distribution, indicating a hint of publication bias. When we eliminated the most unfavorable point (Johannsen et al., 2006), the size of the merger effect was statistically significant [SMD = –0.33, 95% CI (–0.45, –0.20); *p* < 0.00001]. The heterogeneity among most pooled studies was low (*p* = 0.41, *I*^2^= 2%).

When we deleted the most positive outlier (Gault et al., 2016), the size of the merger effect was statistically significant [SMD = –0.26, 95% CI (–0.38, –0.14); *p* < 0.0001]. The heterogeneity among most pooled studies was low (*p* = 0.14, *I*^2^= 37%).

##### Galantamine

Six articles (Raskind et al., 2000; Tariot et al., 2000; Wilcock et al., 2000; Rockwood et al., 2001; Wilkinson and Murray, 2001; Brodaty et al., 2005) used ADAS-cog to assess cognitive changes, and eleven studies reported the changes (Figure 2B). Among the available data, the cognitive effects of all drugs were displayed by meta-analysis, and the pooled SMDs between galantamine and placebo in ADAS-cog was significant [SMD = –0.49, 95% CI (–0.56, –0.43); *p* < 0.00001]. The heterogeneity among most pooled studies was low (*p* = 0.66, *I*^2^= 0%). The funnel plots (data not shown) did not show symmetric distribution, indicating a hint of publication bias. When we eliminated the most unfavorable point (Wilkinson and Murray, 2001), the size of the merger effect was significant [SMD = –0.50, 95% CI (–0.57, –0.44); *p* < 0.00001]. The heterogeneity among most pooled studies was low (*p* = 0.78, *I*^2^= 0%). When we deleted the most positive outlier (Raskind et al., 2000), the size of the merger effect was statistically significant [SMD = –0.47, 95% CI (–0.54, –0.41); *p* < 0.00001]. The heterogeneity among most pooled studies was low (*p* = 0.88, *I*^2^= 0%).

##### Rivastigmine

Four articles (Forette et al., 1999; Rosler et al., 1999; Feldman and Lane, 2007; Winblad et al., 2007) used ADAS-cog to assess cognitive changes (Figure 2C). Significant differences were found between all drugs and the placebo when cognitive effects were calculated by meta-analysis from the available data. The pooled SMDs in the changes between the rivastigmine and placebo groups was significant in ADAS-cog [SMD = –0.65, 95% CI (–1.06, –0.23); *p* = 0.002]. The heterogeneity among most pooled studies was high (*p* < 0.00001, *I*^2^= 92%). The funnel plots (data not shown) did not show symmetric distribution, which indicated a hint of publication bias. When we eliminated the most unfavorable point (Rosler et al., 1999), the size of the merger effect was significant [SMD = –0.96, 95% CI (–1.62, –0.31); *p* = 0.004]. The heterogeneity among most pooled studies was high (*p* < 0.00001, *I*^2^= 95%). When we deleted the most positive outlier (Forette et al., 1999), the size of the merger effect was statistically significant [SMD = –0.29, 95% CI (–0.40, –0.19); *p* < 0.00001]. The heterogeneity among most pooled studies was low(*p* = 0.32, *I*^2^= 13%).

##### Memantine

Four articles (Peskind et al., 2006; Bakchine and Loft, 2008; Porsteinsson et al., 2008; Wang et al., 2013) used ADAS-cog to assess cognitive changes (Figure 2D). No significant difference was found between all drugs and placebo when cognitive effects were calculated by meta-analysis from the available data. The pooled, SMDs between memantine and placebo varied in ADAS-cog [SMD = –0.12, 95% CI (–0.24, –0.01); *p* = 0.03]. The heterogeneity among most pooled studies was low (*p* = 0.20, *I*^2^= 35%). The funnel plots (data not shown) did not show symmetric distribution, which indicated a hint of publication bias. When we eliminated the most unfavorable point (Wang et al., 2013), the size of the merger effect was significant [SMD = –0.14, 95% CI (–0.25, –0.02); *p* = 0.02]. The heterogeneity among most pooled studies was low (*p* = 0.38, *I*^2^= 0%). When we deleted the most positive outlier (Peskind et al., 2006), the size of the merger effect was not statistically significant [SMD = –0.07, 95% CI (–0.21, 0.07); *p* = 0.33]. The heterogeneity among most pooled studies was low (*p* = 0.27, *I*^2^= 23%).

#### Functional Outcome

##### Donepezil

**FIGURE 3:**
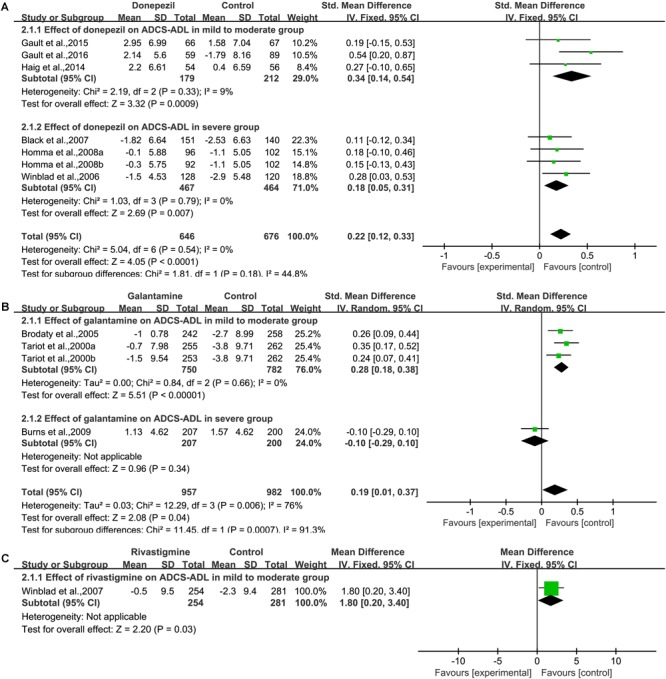
Functional outcomes on the ADCS/ADL subscale (change from baseline) in AD patients in trials of cholinesterase inhibitors, according (**A**, Donepezil; **B**, Galantamine; **C**, Rivastigmine) to drug and dose.

We pooled the data of AD Cooperative Study Activities of Daily Living Inventory from six articles (Winblad et al., 2006; Black et al., 2007; Homma et al., 2008; Haig et al., 2014; Gault et al., 2015, 2016) (Figure 3A), and significant benefit was found with donepezil treatment [SMD = 0.22, 95% CI (0.12, 0.33); *p* < 0.0001]. The heterogeneity among most pooled studies was low (*p* = 0.54, *I*^2^= 0%). The funnel plots (data not shown) did not show symmetric distribution, which indicated a hint of publication bias. When we deleted the most positive outlier (Gault et al., 2016), the merged effect size was also statistically significant [SMD = 0.19, 95% CI (0.07, 0.30); *p* = 0.001]. The heterogeneity among most pooled studies was low (*p* = 0.94, *I*^2^ = 0%). When we eliminated the most unfavorable point (Black et al., 2007), the merged effect size was significant [SMD = 0.26, 95% CI (0.14, 0.38); *p* < 0.0001]. The heterogeneity among most pooled studies was low (*p* = 0.59, *I*^2^= 0%).

##### Galantamine

We pooled the data of AD Cooperative Study Activities of Daily Living Inventory from three articles (Tariot et al., 2000; Brodaty et al., 2005; Burns et al., 2009) (Figure 3B), and significant benefit was found with galantamine treatment [SMD = 0.19, 95% CI (0.01, 0.37); *p* = 0.04]. The heterogeneity among most pooled studies was high (*p* = 0.006, *I*^2^= 76%). The funnel plots (data not shown) did not show symmetric distribution, which indicated a hint of publication bias. When we deleted the most positive outlier (Tariot et al., 2000), the merged effect size was not significant [SMD = 0.14, 95% CI (–0.08, 0.36); *p* = 0.21]. The heterogeneity among most pooled studies was high (*p* = 0.01, *I*^2^= 77%). When we eliminated the most unfavorable point (Burns et al., 2009), the merged effect size was also statistically significant [SMD = 2.21, 95% CI (1.42, 2.99); *p* < 0.00001]. The heterogeneity among most pooled studies was low (*p* = 0.34, *I*^2^= 6%).

##### Rivastigmine

We pooled the data of AD Cooperative Study Activities of Daily Living Inventory from one studies (Winblad et al., 2007) (Figure 3C), and significant benefit was found with rivastigmine treatment [MD = 1.80, 95% CI (0.20, 3.40); *p* = 0.03].

##### Memantine

We pooled the data of AD Cooperative Study Activities of Daily Living Inventory 19 (ADCS-ADL_19_) from four studies (Reisberg et al., 2003; Tariot et al., 2004; van Dyck et al., 2007; Grossberg et al., 2013) (Supplementary Figure S1A) and significant benefit was found with memantine treatment [SMD = 0.15, 95% CI (0.05, 0.24); *p* = 0.003]. The heterogeneity among most pooled studies was low (*p* = 0.39, *I*^2^= 7%). The funnel plots (data not shown) did not show a fairly symmetric distribution, which indicated no hint of publication bias. When we deleted the most positive outlier (Reisberg et al., 2003), the merged effect size was significant [SMD = 0.12, 95% CI (0.01, 0.22); *p* = 0.03]. The heterogeneity among most pooled studies was low (*p* = 0.64, *I*^2^= 0%). When we eliminated the most unfavorable point (Grossberg et al., 2013), the merged effect size was significant [SMD = 0.19, 95% CI (0.06, 0.32); *p* = 0.003]. The heterogeneity among most pooled studies was low (*p* = 0.39, *I*^2^= 0%).

In a pooled study of Activities of Daily Living Inventory 23 (ADCS-ADL_23_), the pooled analysis of both studies showed that no significant benefit was found with memantine treatment [SMD = 0.00, 95% CI (–0.11, 0.12); *p* = 0.93] (Supplementary Figure S1B). The heterogeneity among most pooled studies was low (*p* = 0.99, *I*^2^= 0%). The funnel plots (data not shown) did not show a symmetric distribution, which indicated no hint of publication bias.

#### Behavioral Outcome

##### Donepezil

**FIGURE 4:**
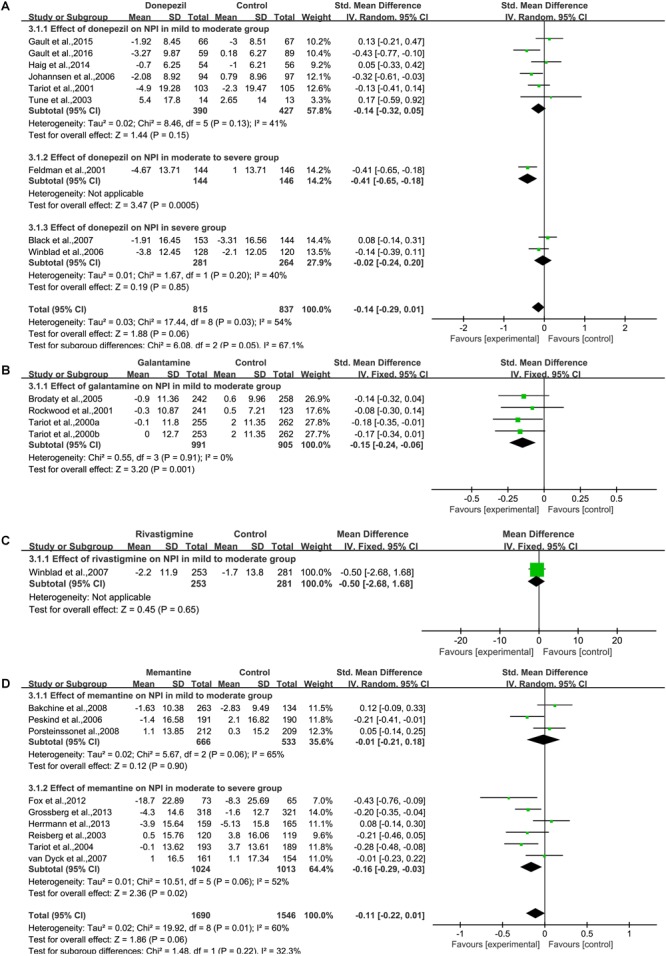
Behavior outcomes on the NPI scale (change from baseline) in AD patients in trials on cholinesterase inhibitors (**A**, Donepezil; **B**, Galantamine; **C**, Rivastigmine) and memantine **(D)**, according to drug and dose.

We pooled NPI data from nine studies (Feldman et al., 2001; Tariot et al., 2001; Tune et al., 2003; Johannsen et al., 2006; Winblad et al., 2006; Black et al., 2007; Haig et al., 2014; Gault et al., 2015, 2016) (Figure 4A), which contained detailed information on the baseline and final observation times for donepezil and placebo. Donepezil treatment showed no significant effect on the behavioral outcome of NPI assessment [SMD = –0.14, 95% CI (–0.29, 0.01); *p* = 0.06]. The heterogeneity among most pooled studies was moderate (*p* = 0.03, *I*^2^= 54%). The funnel plots (data not shown) did not show symmetric distribution, which indicated a hint of publication bias. When we deleted the most positive outlier (Gault et al., 2016), the merged effect size was not significant [SMD = –0.11, 95% CI (–0.26, 0.04); *p* = 0.15]. The heterogeneity among most pooled studies was moderate (*p* = 0.04, *I*^2^= 51%). When we eliminated the most unfavorable point (Tune et al., 2003), the merged effect size was not significant [SMD = –0.15, 95% CI (–0.31, 0.00); *p* = 0.05]. The heterogeneity among most pooled studies was moderate (*p* = 0.02, *I*^2^= 58%).

##### Galantamine

We pooled NPI data from three articles (Tariot et al., 2000; Rockwood et al., 2001; Brodaty et al., 2005) (Figure 4B), which contained detailed information on the baseline and final observation times for galantamine and placebo. Galantamine treatment showed a significant effect on the behavioral outcome when assessed by NPI [SMD = –0.15, 95% CI (–0.24, –0.06); *p* = 0.001]. The heterogeneity among most pooled studies was low (*p* = 0.91, *I*^2^= 0%). The funnel plots (data not shown) did not show a symmetric distribution, which indicated no hint of publication bias.

##### Rivastigmine

We pooled NPI data from one study (Winblad et al., 2007) (Figure 4C), which contained detailed information on the baseline and final observation times for rivastigmine and placebo. Rivastigmine treatment showed no significant effect on behavioral outcome when assessed by NPI [MD = –0.50, 95% CI (–2.68, 1.68); *p* = 0.65].

##### Memantine

We pooled NPI data from nine studies (Reisberg et al., 2003; Tariot et al., 2004; Peskind et al., 2006; van Dyck et al., 2007; Bakchine and Loft, 2008; Porsteinsson et al., 2008; Fox et al., 2012; Grossberg et al., 2013; Herrmann et al., 2013), which contained detailed information on the baseline and final observation times for memantine and placebo (Figure 4D). Memantine treatment showed no significant effect on the behavioral outcomes when assessed by NPI [SMD = –0.11, 95% CI (–0.22, 0.01); *p* = 0.06]. The heterogeneity among most pooled studies was moderate (*p* = 0.01, *I*^2^= 60%). The funnel plots (data not shown) did not show symmetric distribution, indicating hint of publication bias. When we eliminated the most unfavorable point (Herrmann et al., 2013), the size of the merged effect is an important influence size [SMD = –0.13, 95% CI (–0.25, –0.01); *p* = 0.03]. The heterogeneity among most pooled studies was moderate (*p* = 0.02, *I*^2^= 59%). When we deleted the most positive outlier (Fox et al., 2012), the merged effect size was not significant [SMD = –0.08, 95% CI (–0.19, –0.03); *p* = 0.14]. The heterogeneity among most pooled studies was moderate (*p* = 0.02, *I*^2^= 57%).

#### Global Assessment

##### Donepezil

**FIGURE 5:**
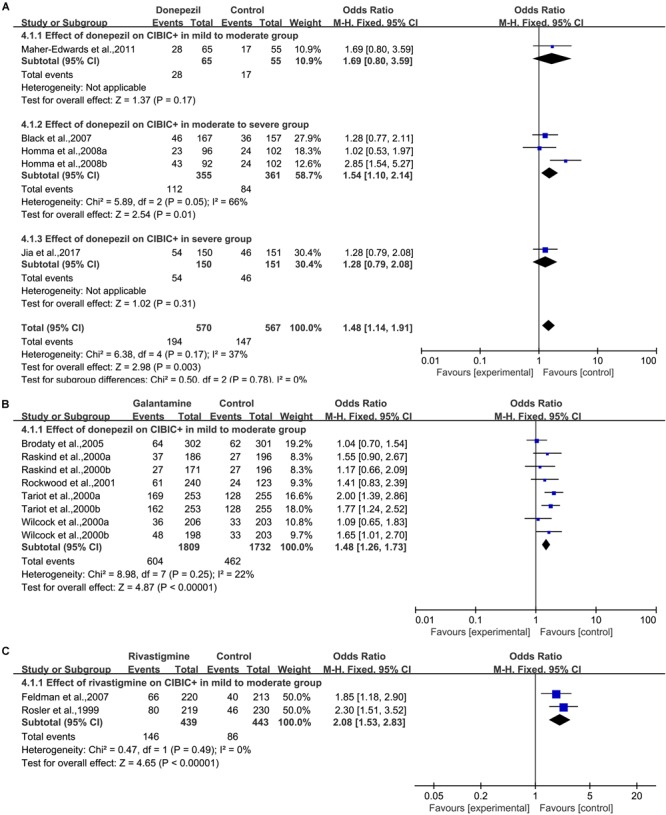
Global change outcomes in AD patients in cholinesterase inhibitors (**A**, Donepezil; **B**, Galantamine; **C**, Rivastigmine) trials based on CIBIC+ versus no change or worsening compared to the baseline according to drug and dose.

Four articles (Black et al., 2007; Homma et al., 2008; Maher-Edwards et al., 2011; Jia et al., 2017) based on interviews and CIBIC+ used the clinician’s impression of change to evaluate the clinician’s global impression (Figure 5A). We pooled the results from the studies and found a significant difference in the donepezil group [OR = 1.48, 95% CI (1.14, 1.91); *p* = 0.003]. The heterogeneity among most pooled studies was low (*p* = 0.17, *I*^2^= 37%). The funnel plots (data not shown) did not show symmetric distribution, which indicated a hint of publication bias. When we eliminated the most unfavorable point (Homma et al., 2008), the merged effect size was a significant OR = 1.58, 95% CI (1.19, 2.09); *p* = 0.001]. The heterogeneity among most pooled studies was moderate (*p* = 0.17, *I*^2^= 40%). When we deleted the most positive outlier (Homma et al., 2008), the merged effect size was not significant [OR = 1.28, 95% CI (0.96, 1.70); *p* = 0.09]. The heterogeneity among most pooled studies was low (*p* = 0.81, *I*^2^= 0%).

##### Galantamine

Five articles (Raskind et al., 2000; Tariot et al., 2000; Wilcock et al., 2000; Rockwood et al., 2001; Brodaty et al., 2005) based on interviews and CIBIC+ used the clinician’s impression of change to evaluate the clinician’s global impression (Figure 5B). We pooled the results from the studies and found a significant difference in the galantamine group [OR = 1.48, 95% CI (1.26, 1.73); *p* < 0.00001]. The heterogeneity among most pooled studies was low (*p* = 0.25, *I*^2^= 22%). The funnel plots (data not shown) did not show symmetric distribution, indicating a hint of publication bias. When we eliminated the most unfavorable point (Brodaty et al., 2005), the merged effect size was also statistically significant [OR = 1.59, 95% CI (1.33, 1.88); *p* < 0.00001]. The heterogeneity among most pooled studies was low (*p* = 0.52, *I*^2^= 0%). When we deleted the most positive outlier (Tariot et al., 2000), the merged effect size was significant [OR = 1.38, 95% CI (1.16, 1.64); *p* = 0.0004]. The heterogeneity among most pooled studies was low (*p* = 0.46, *I*^2^= 0%).

##### Rivastigmine

Two articles (Rosler et al., 1999; Feldman and Lane, 2007) based on interviews and CIBIC+ used the clinician’s impression of change to evaluate the clinician’s global impression (Figure 5C). We pooled the results of studies and found a significant difference in the rivastigmine group [OR = 2.08, 95% CI (1.53, 2.83); *p* < 0.00001]. The heterogeneity among most pooled studies was low (*p* = 0.49, *I*^2^= 0%). The funnel plots (data not shown) did not show a symmetric distribution, indicating no hint of publication bias.

##### Memantine

Two articles (Tariot et al., 2004; Bakchine and Loft, 2008) based on interviews and CIBIC+ used the clinician’s impression of change to evaluate the clinician’s global impression (Supplementary Figure S1C). We pooled the results from the studies, and no significant difference was found in the memantine group [OR = 1.23, 95% CI (0.85, 1.78); *p* = 0.28]. The heterogeneity among most pooled studies was low (*p* = 0.70, *I*^2^= 0%). The funnel plots (data not shown) did not show a symmetric distribution, which indicated no hint of publication bias.

### Safety and Tolerability

#### Donepezil

**FIGURE 6:**
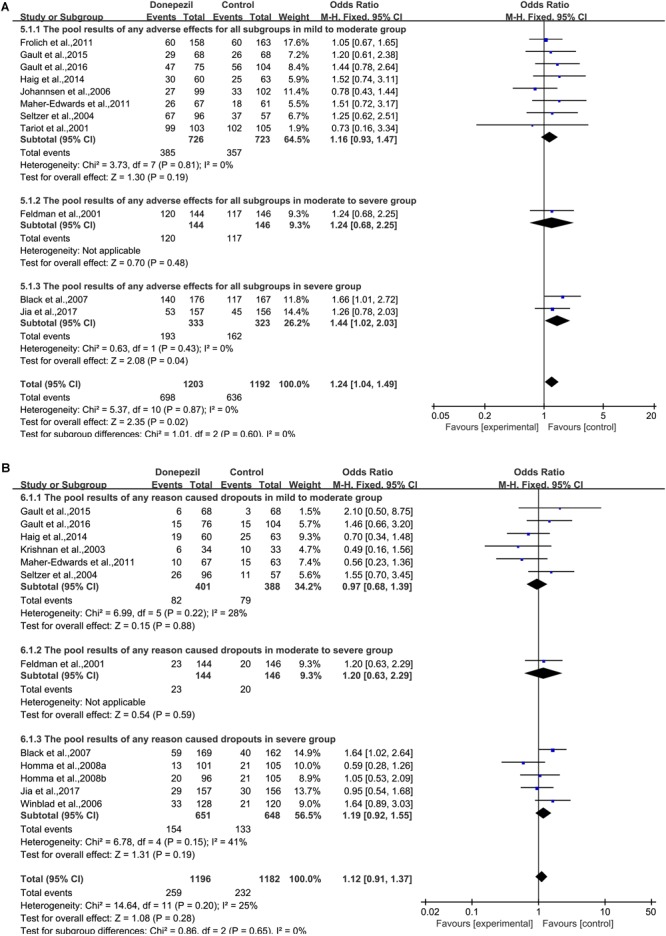
Safety and tolerability outcome comparison for any adverse effect **(A)** and any reason that caused dropouts **(B)** in the donepezil group versus the placebo group.

When any AE was considered, there was a statistically significant association between the donepezil group and the placebo group [OR = 1.24, 95% CI (1.04, 1.49); *p* = 0.02] (Figure 6A). The heterogeneity among most pooled studies was low (*p* = 0.87, *I*^2^= 0%). The funnel plots (data not shown) did not show a symmetric distribution, indicating no hint of publication bias. Overall, when considering the number of dropouts caused by any reason, no difference was found between patients treated with donepezil and placebo [OR = 1.12, 95% CI (0.91, 1.37); *p* = 0.28] (Figure 6B). The heterogeneity among most pooled studies was low (*p* = 0.20, *I*^2^= 25%). The funnel plots (data not shown) did not show symmetric distribution, which indicated a hint of publication bias. When we deleted the greatest positive outlier (Gault et al., 2015), the merged effect size was not statistically significant [OR = 1.10, 95% CI (0.90, 1.35); *p* = 0.35]. The heterogeneity among most pooled studies was low (*p* = 0.18, *I*^2^= 28%). When we eliminated the most unfavorable point (Krishnan et al., 2003), the merged effect size was not significant [OR = 1.15, 95% CI (0.94, 1.41); *p* = 0.19]. The heterogeneity among most pooled studies was low (*p* = 0.24, *I*^2^= 21%). When the number of dropouts was caused by adverse effects, a significant difference was found between the donepezil group and the placebo group [OR = 1.58, 95% CI (1.22, 2.05); *p* = 0.0006] (Supplementary Figure S2). The heterogeneity among most pooled studies was low (*p* = 0.80, *I*^2^= 0%). The funnel plots (data not shown) did not show a fairly symmetric distribution, which indicated no hint of publication bias.

#### Galantamine

When any AE was considered, there was a statistically significant association between the galantamine group and the placebo group [OR = 1.84, 95% CI (1.41, 2.41); *p* < 0.00001] (Supplementary Figure S3A). The heterogeneity among most pooled studies was high (*p* = 0.0001, *I*^2^= 70%). The funnel plots (data not shown) did not show symmetric distribution, which indicated a hint of publication bias. When we eliminated the most unfavorable point (Burns et al., 2009), the merged effect size was significant [OR = 1.94, 95% CI (1.47, 2.55); *p* < 0.00001]. The heterogeneity among most pooled studies was high (*p* = 0.0002, *I*^2^= 70%). When we deleted the greatest positive outlier (Rockwood et al., 2001), the merged effect size was significant [OR = 1.71, 95% CI (1.33, 2.20); *p* < 0.0001]. The heterogeneity among most pooled studies was moderate (*p* = 0.003, *I*^2^= 63%). Overall, when the number of dropouts caused by any reason was considered, significant difference was found between patients treated with galantamine and placebo [OR = 1.95, 95% CI (1.52, 2.50); *p* < 0.00001] (Supplementary Figure S3B). The heterogeneity among most pooled studies was moderate (*p* = 0.002, *I*^2^= 63%). The funnel plots (data not shown) did not show symmetric distribution, which indicated a hint of publication bias. When we eliminated the most unfavorable point (Burns et al., 2009), the merged effect size was significant [OR = 2.08, 95% CI (1.64, 2.63); *p* < 0.00001]. The heterogeneity among most pooled studies was moderate (*p* = 0.01, *I*^2^= 55%). When we deleted the greatest positive outlier (Wilkinson and Murray, 2001), the merged effect size was significant [OR = 1.84, 95% CI (1.45, 2.33); *p* < 0.00001]. The heterogeneity among most pooled studies was moderate (*p* = 0.008, *I*^2^= 58%). When the number of dropouts caused by adverse effects was considered, a significant difference was found between the galantamine group and the placebo group [OR = 2.48, 95% CI (1.64, 3.75); *p* < 0.0001] (Supplementary Figure S4). The heterogeneity among most pooled studies was high (*p* < 0.00001, *I*^2^= 77%). The funnel plots (data not shown) did not show symmetric distribution, which indicated a hint of publication bias. When we deleted the greatest positive outlier (Rockwood et al., 2001), the merged effect size was significant [OR = 2.26, 95% CI (1.50, 3.40); *p* < 0.0001]. The heterogeneity among most pooled studies was high (*p* < 0.0001, *I*^2^= 76%). When we eliminate the most unfavorable point (Burns et al., 2009), the merged effect size was significant [OR = 2.73, 95% CI (1.83, 4.09); *p* < 0.00001]. The heterogeneity among most pooled studies was high (*p* < 0.0001, *I*^2^= 73%).

#### Rivastigmine

Overall, when the number of dropouts caused by any reason was considered, a difference between patients treated with rivastigmine and placebo was found [OR = 2.24, 95% CI (1.29, 3.88); *p* = 0.004] (Supplementary Figure S5A). The heterogeneity among most pooled studies was moderate (*p* = 0.03, *I*^2^= 66%). The funnel plots (data not shown) did not show symmetric distribution, which indicated a hint of publication bias. When we eliminated the most unfavorable point (Feldman and Lane, 2007), the merged effect size was significant [OR = 2.64, 95% CI (1.93, 3.62); *p* < 0.00001]. The heterogeneity among most pooled studies was moderate (*p* = 0.13, *I*^2^= 51%). When we deleted the greatest positive outlier (Forette et al., 1999), the effect size was significant [OR = 2.04, 95% CI (1.20, 3.45); *p* = 0.008]. The heterogeneity among most pooled studies was moderate (*p* = 0.04, *I*^2^= 69%). When the number of dropouts caused by adverse effects was concerned, a significant difference was found between the rivastigmine group and the placebo group [OR = 2.38, 95% CI (1.12, 5.02); *p* = 0.02] (Supplementary Figure S5B). The heterogeneity among most pooled studies was high (*p* = 0.01, *I*^2^= 72%). The funnel plots (data not shown) did not show symmetric distribution, which indicated a hint of publication bias. When we eliminated the most unfavorable point (Feldman and Lane, 2007), the merged effect size was significant [OR = 3.04, 95% CI (1.99, 4.65); *p* < 0.00001]. The heterogeneity among most pooled studies was moderate (*p* = 0.07, *I*^2^= 63%). When we deleted the greatest positive outlier (Forette et al., 1999), the effect size was not significant [OR = 2.03, 95% CI (0.97, 4.27); *p* = 0.06]. The heterogeneity among most pooled studies was high (*p* = 0.01, *I*^2^= 76%).

#### Memantine

When any AE was considered, no statistically significant association was found between the memantine group and the placebo group [OR = 0.97, 95% CI (0.92, 1.02); *p* = 0.28] (Supplementary Figure S6A). The heterogeneity among most pooled studies was moderate (*p* = 0.02, *I*^2^= 64%). The funnel plots (data not shown) did not show symmetric distribution, which indicated a hint of publication bias. When we eliminated the most unfavorable point (Peskind et al., 2006), the merged effect size was not significant [OR = 0.96, 95% CI (0.91, 1.01); *p* = 0.09]. The heterogeneity among most pooled studies was moderate (*p* = 0.02, *I*^2^= 66%). When we deleted the greatest positive outlier (Porsteinsson et al., 2008), the merged effect size was not significant [OR = 1.01, 95% CI (0.96, 1.07); *p* = 0.62]. The heterogeneity among most pooled studies was low (*p* = 0.77, *I*^2^= 0%). Overall, when the number of dropouts caused by any reason was concerned, no difference was found between patients treated with memantine and placebo [OR = 0.93, 95% CI (0.79, 1.11); *p* = 0.44] (Supplementary Figure S6B). The heterogeneity among most pooled studies was low (*p* = 0.13, *I*^2^= 35%). The funnel plots (data not shown) did not show a fairly symmetric distribution, which indicated no hint of publication bias. When we eliminated the most unfavorable point (Bakchine and Loft, 2008), the merged effect size was not significant [OR = 0.88, 95% CI (0.74, 1.06); *p* = 0.18]. The heterogeneity among most pooled studies was low (*p* = 0.26, *I*^2^= 21%). When we deleted the greatest positive outlier (Tariot et al., 2004), the effect size was not significant [OR = 1.02, 95% CI (0.85, 1.22); *p* = 0.86]. The heterogeneity among most pooled studies was low (*p* = 0.48, *I*^2^= 0%). When the number of dropouts caused by adverse effects was concerned, no significant difference was found between the memantine group and the placebo group [OR = 1.24, 95% CI (0.97, 1.58); *p* = 0.08] (Supplementary Figure S7). The heterogeneity among most pooled studies was moderate (*p* = 0.07, *I*^2^= 44%). The funnel plots (data not shown) did not show symmetric distribution, which indicated a hint of publication bias. When we eliminated the most unfavorable point (Tariot et al., 2004), the merged effect size was significant [OR = 1.40, 95% CI (1.08, 1.83); *p* = 0.01]. The heterogeneity among most pooled studies was low (*p* = 0.31, *I*^2^= 16%). When we deleted the greatest positive outlier (Bakchine and Loft, 2008), the merged effect size was not significant [OR = 1.17, 95% CI (0.91, 1.50); *p* = 0.23]. The heterogeneity among most pooled studies was moderate (*p* = 0.09, *I*^2^= 43%).

Among these side effects, some gastrointestinal and nervous system side effects such as nausea, vomiting, diarrhea, anorexia, dizziness, depression and headache were observed. Since some studies did not report these events in detail, we did not separately compare the incidence of AEs.

## Discussion

Different methods of measurement were used in assessing treatment outcomes. We divided the impact into four parts, cognitive function, functional outcome, behavioral outcome, and global assessment. In this article, we presented a meta-analysis of the effects of donepezil, galantamine, rivastigmine, and memantine (Supplementary Table 1) on mild-to-moderate, moderate-to-severe, and severe AD using the ADAS-cog (cognitive function), ADCS-ADL (functional outcome), NPI (behavioral outcome) and CIBIC+ scores (global assessment). We obtained data from 6611 AD patients across 36 trials. Meta-analyses of these placebo-controlled trials showed that there were significant differences between the donepezil and placebo groups using ADAS-cog, ADCS-ADL, and CIBIC+, between the galantamine and placebo groups using ADAS-cog, ADCS-ADL, NPI, and CIBIC+, between the rivastigmine and placebo groups using ADAS-cog, ADCS-ADL, and CIBIC+, and between the memantine and placebo groups using ADAS-cog, and ADCS-ADL_19_ (Supplementary Table 6). This observation suggests that donepezil is beneficial for stabilizing or slowing the decline in cognitive function, functional outcome, and global assessment change in AD patients. Analysis of the entire database showed consistent results, which indicated positive results with the donepezil treatment and improvement in the condition of mild-to-moderate, moderate-to-severe, and severe AD patients. To our knowledge, this is the first meta-analysis to reveal the favorable performance of donepezil with mild-to-moderate, moderate-to-severe, and severe AD. This observation suggests that galantamine is beneficial for stabilizing or slowing the decline in cognitive function, functional outcome, behavior outcome and global assessment change in AD patients. To our knowledge, this is the first meta-analysis to reveal the favorable performance of galantamine with mild-to-moderate, moderate-to-severe, and severe AD. This observation suggests that rivastigmine is beneficial for stabilizing or slowing the decline in cognitive function, functional outcome, and global assessment change in AD patients. Analysis of the entire database showed consistent results, which indicated positive results with rivastigmine treatment and improvement in the condition of mild-to-moderate, moderate-to-severe, and severe AD patients. There was no significant effect on the outcome of NPI-assessed behavioral therapy, except galantamine. This observation suggests that memantine is beneficial for stabilizing or slowing the decline in cognitive function, and functional outcome change in AD patients. However, there was no significant effect according to the ADCS-ADL_23_, NPI, and CIBIC+ tests, which indicated that memantine treatment has no significant effect on these cognitive aspects of AD patients.

Safety and tolerability are as important as the effectiveness of the interventions in clinical research. In the current meta-analysis, when any AE, dropout caused by any reason, or adverse effect was considered in all patients or subgroups, significant differences were found between the galantamine or rivastigmine groups and the placebo group, which indicated that donepezil and galantamine were not sufficiently safe and tolerable for AD treatment. However, no significant difference was found in all patients or subgroups when the donepezil group and the placebo group were compared, which indicates that donepezil is safe and tolerable enough for AD treatment. When any AE, dropout caused by any reason, or adverse effect was considered, no significant difference was found between the memantine group and the placebo group in all patients or subgroups, which indicates that memantine is safe and tolerable enough for AD treatment. Doody et al. (2007) reported a lower rate of discontinuation due to AEs in the memantine group and a lower discontinuation rate than the placebo group. On the other hand, Yang et al. (2013) and Jiang and Jiang (2015) did not find the differences.

Compared with the published meta-analysis (Loveman et al., 2006; Raina et al., 2008; Bond et al., 2012; Di Santo et al., 2013), galantamine has potent therapeutic effects on all aspects of the treatment of AD, but the other three drugs do not have effective therapeutic effects on some aspects (Supplementary Table 6). Therefore, based on the current statistical conclusion, we have determined that we prefer galantamine for the treatment of AD. However, due to limited data, we should also consider additional data to obtain more stable results.

This analysis is subject to the number of limitations. First, although all studies are short-lived, the duration of the study is still a variable and may be a factor to consider. Second, the results of neuropsychiatric symptoms are based on a relatively small number of trials. This can affect the wider range of estimates. Third, meta-analysis data come only from published scientific literature, and some negative results and non-statistical data are difficult to publish; therefore, there is publication bias. Fourth, the genetic backgrounds of these patients involved in our analysis were different, which could potentially influence the rivastigmine treatment. Finally, some of our trials used flexible drug doses. Overall, the results indicate benefits in cognition but the efficacy on functional, behavioral, and global change symptoms is questionable in patients with mild to moderate to severe AD. Our results might suggest a possible perspective for anti-dementia drug trials, such as increasing placebo effects over time and heterogeneity of neuropsychiatric symptoms in AD. The results of this study still need to be confirmed by further studies.

## Conclusion

Our analysis is the first attempt to incorporate available direct or indirect evidence to evaluate the efficacy and safety of 4 drugs in the treatment of AD. The results suggest that donepezil exhibited a significant positive efficacy with respect to cognition, function, behavior, and global change. However, the efficacy of rivastigmine or galantamine in behavioral outcome is questionable in patients with mild to moderate to severe AD. The efficacy of memantine on global assessment is questionable in patients with mild-to-moderate and moderate-to-severe AD. This review shows that donepezil, galantamine, rivastigmine, and memantine can delay cognitive impairment in patients with mild-to-moderate-to-severe AD for at least 52 weeks. Based on the current statistical conclusions, galantamine is effective in treating all aspects of AD and may be the first choice in the treatment of AD. However, due to limited data, we should also consider additional data to obtain more stable results.

## Author Contributions

WZ and PZ designed the study. D-DL performed the experiments and data analysis with the help of Y-HZ, WZ, and PZ. D-DL, WZ, and PZ wrote the manuscript.

## Conflict of Interest Statement

The authors declare that the research was conducted in the absence of any commercial or financial relationships that could be construed as a potential conflict of interest.
