# Supplementary material for: Meta-Analysis of Randomized Controlled Trials on the Efficacy and Safety of Donepezil, Galantamine, Rivastigmine, and Memantine for the Treatment of Alzheimer’s Disease
Source: Front Neurosci. 2019 May 15;13:472. doi: 10.3389/fnins.2019.00472 (PMC6529534; doi:10.3389/fnins.2019.00472)
Supplement: Supplementary file 1 [file Data_Sheet_1.PDF]

A

Study or Subgroup	Experimental Mean	Experimental SD	Experimental Total	Control Mean	Control SD	Control Total	Weight	Std. Mean Difference IV, Fixed, 95% CI
Grossberg et al.,2013	-0.7	6.9	331	-1.3	7.7	328	40.4%	0.08 [-0.07, 0.23]
Reisberg et al.,2003	-3.1	6.79	124	-5.2	6.33	123	15.0%	0.32 [0.07, 0.57]
Tariot et al.,2004	-2	7.04	198	-3.4	7.16	197	24.1%	0.20 [-0.00, 0.39]
van Dyck et al.,2007	-2	7.85	171	-2.7	7.71	165	20.6%	0.09 [-0.12, 0.30]
**Total (95% CI)**			**824**			**813**	**100.0%**	**0.15 [0.05, 0.24]**

Heterogeneity: Chi² = 3.02, df = 3 (P = 0.39); I² = 1%
Test for overall effect: Z = 2.96 (P = 0.003)

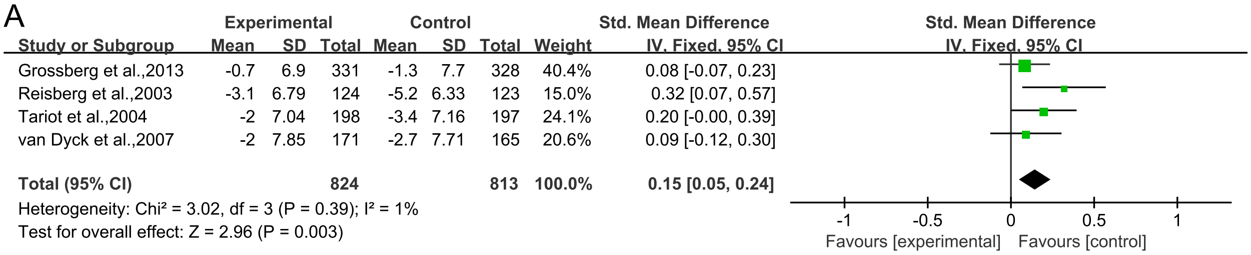

B

Study or Subgroup	Favours [experimental] Mean	Favours [experimental] SD	Favours [experimental] Total	Control Mean	Control SD	Control Total	Weight	Std. Mean Difference IV, Fixed, 95% CI
Bakchine et al.,2008	-1.98	9.15	267	-2.1	8.57	134	30.3%	0.01 [-0.19, 0.22]
Peskind et al.,2006	-2.9	10.78	196	-3	10.83	198	33.4%	0.01 [-0.19, 0.21]
Porsteinssonet al.,2008	-2.9	16.66	214	-2.8	15.92	213	36.3%	-0.01 [-0.20, 0.18]
**Total (95% CI)**			**677**			**545**	**100.0%**	**0.00 [-0.11, 0.12]**

Heterogeneity: Chi² = 0.02, df = 2 (P = 0.99); I² = 0%
Test for overall effect: Z = 0.08 (P = 0.93)

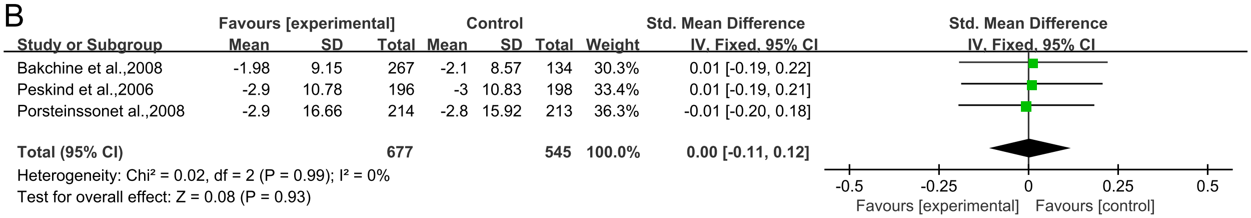

C

Study or Subgroup	Memantine Events	Memantine Total	Control Events	Control Total	Weight	Odds Ratio M-H, Fixed, 95% CI
**4.1.1 Effect of memantine on CIBIC+ in mild to moderate group**						
Bakchine et al.,2008	73	268	33	135	62.0%	1.16 [0.72, 1.86]
**Subtotal (95% CI)**		**268**		**135**	**62.0%**	**1.16 [0.72, 1.86]**
Total events	73		33			
Heterogeneity: Not applicable						
Test for overall effect: Z = 0.60 (P = 0.55)						
**4.1.2 Effect of memantine on CIBIC+ in moderate to severe group**						
Tariot et al.,2004	30	198	23	196	38.0%	1.34 [0.75, 2.41]
**Subtotal (95% CI)**		**198**		**196**	**38.0%**	**1.34 [0.75, 2.41]**
Total events	30		23			
Heterogeneity: Not applicable						
Test for overall effect: Z = 0.99 (P = 0.32)						
**Total (95% CI)**		**466**		**331**	**100.0%**	**1.23 [0.85, 1.78]**
Total events	103		56			

Heterogeneity: Chi² = 0.15, df = 1 (P = 0.70); I² = 0%
Test for overall effect: Z = 1.09 (P = 0.28)
Test for subgroup differences: Chi² = 0.15, df = 1 (P = 0.70), I² = 0%

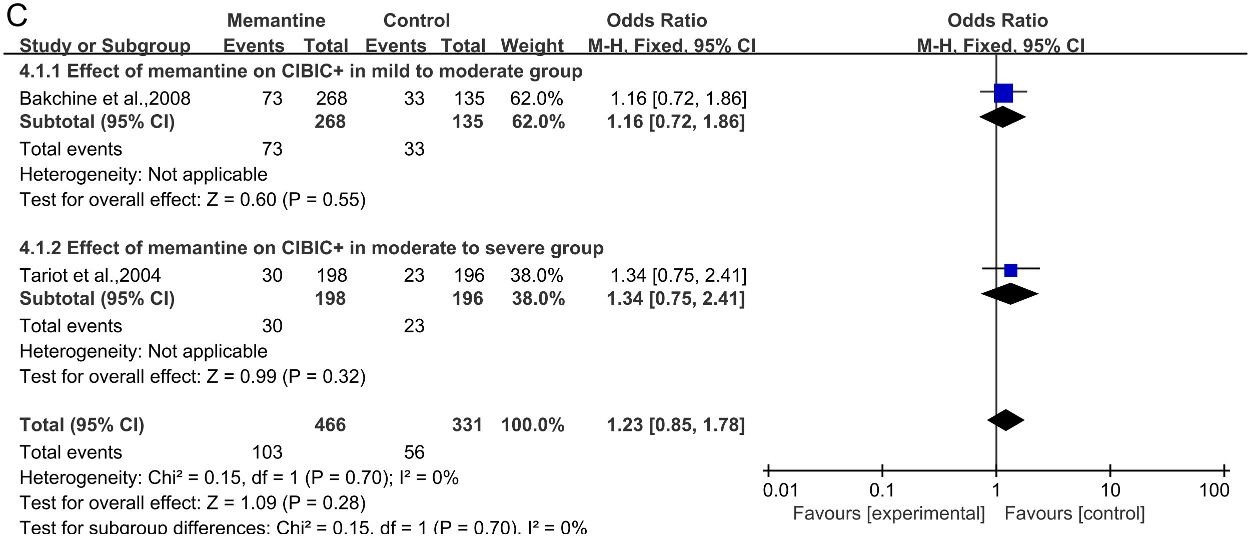

Figure S1: (A,B) Functional outcomes on the ADCS/ADL19 (A) and ADCS/ADL23 (B) subscale (change from baseline) in AD patients in memantine trials by drug and dose. (C) Global change outcomes in AD patients in memantine trials based on CIBIC+ versus no change or worsening compared to baseline by drug and dose.

Study or Subgroup	Donepezil Events	Donepezil Total	Control Events	Control Total	Weight	Risk Ratio M-H, Fixed, 95% CI
**7.1.1 The pool results of adverse effects caused dropouts in mild to moderate group**						
Gault et al.,2015	5	68	2	68	2.4%	2.50 [0.50, 12.44]
Gault et al.,2016	7	76	3	104	3.0%	3.19 [0.85, 11.95]
Haig et al.,2014	4	60	3	63	3.5%	1.40 [0.33, 6.00]
Krishnan et al.,2003	0	34	1	33	1.8%	0.32 [0.01, 7.68]
Maher-Edwards et al.,2011	4	67	2	62	2.5%	1.85 [0.35, 9.75]
Seltzer et al.,2004	15	96	5	57	7.4%	1.78 [0.68, 4.64]
**Subtotal (95% CI)**		**401**		**387**	**20.6%**	**1.89 [1.07, 3.32]**
Total events	35		16			
Heterogeneity: Chi² = 2.10, df = 5 (P = 0.84); I² = 0%						
Test for overall effect: Z = 2.20 (P = 0.03)						
**7.1.2 The pool results of adverse effects caused dropouts in moderate to severe group**						
Feldman et al.,2001	12	144	9	146	10.6%	1.35 [0.59, 3.11]
**Subtotal (95% CI)**		**144**		**146**	**10.6%**	**1.35 [0.59, 3.11]**
Total events	12		9			
Heterogeneity: Not applicable						
Test for overall effect: Z = 0.71 (P = 0.48)						
**7.1.3 The pool results of adverse effects caused dropouts in severe group**						
Black et al.,2007	34	169	18	162	21.8%	1.81 [1.07, 3.07]
Homma et al.,2008a	8	101	11	105	12.8%	0.76 [0.32, 1.80]
Homma et al.,2008b	13	96	11	105	12.5%	1.29 [0.61, 2.75]
Jia et al.,2017	14	157	10	156	11.9%	1.39 [0.64, 3.04]
Winblad et al.,2006	20	128	8	120	9.8%	2.34 [1.07, 5.12]
**Subtotal (95% CI)**		**651**		**648**	**68.8%**	**1.52 [1.11, 2.08]**
Total events	89		58			
Heterogeneity: Chi² = 4.31, df = 4 (P = 0.37); I² = 7%						
Test for overall effect: Z = 2.64 (P = 0.008)						
**Total (95% CI)**		**1196**		**1181**	**100.0%**	**1.58 [1.22, 2.05]**
Total events	136		83			
Heterogeneity: Chi² = 7.00, df = 11 (P = 0.80); I² = 0%						
Test for overall effect: Z = 3.45 (P = 0.0006)						
Test for subgroup differences: Chi² = 0.56, df = 2 (P = 0.75), I² = 0%						

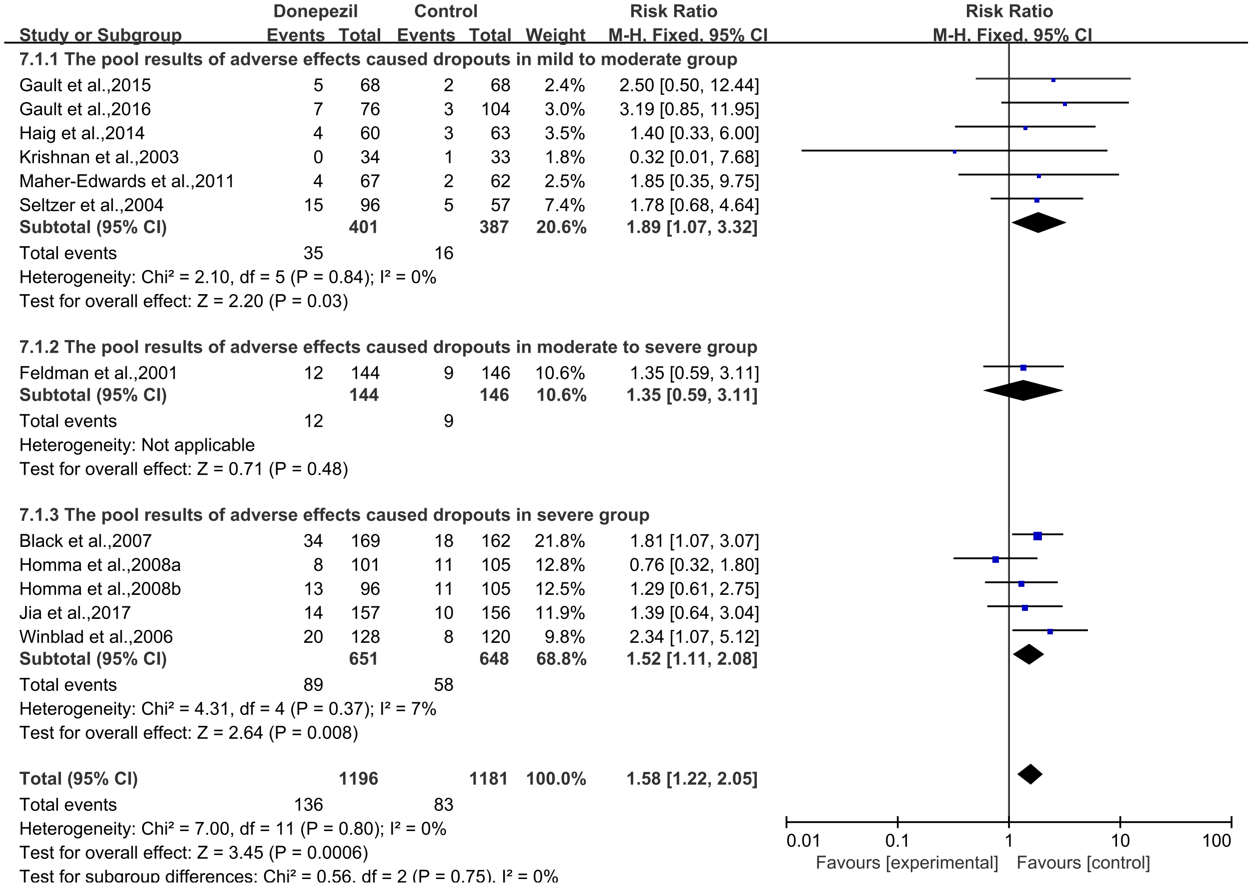

Figure S2: Safety and tolerability outcomes comparison on adverse effects caused dropouts in the donepezil group versus the placebo group.

A

Study or Subgroup	Galantamine Events	Galantamine Total	Control Events	Control Total	Weight	Odds Ratio M-H, Random, 95% CI
**5.1.1 The pool results of any adverse effects for all subgroups in mild to moderate group**						
Brodaty et al.,2005	235	326	224	320	10.3%	1.11 [0.79, 1.56]
Raskind et al.,2000a	195	212	168	213	7.7%	3.07 [1.69, 5.57]
Raskind et al.,2000b	195	211	168	213	7.6%	3.26 [1.78, 5.99]
Rockwood et al.,2001	225	261	79	125	8.6%	3.64 [2.19, 6.04]
Tariot et al.,2000a	206	279	206	286	10.0%	1.10 [0.76, 1.59]
Tariot et al.,2000b	219	273	206	286	9.8%	1.57 [1.06, 2.34]
Wilcock et al.,2000a	182	220	165	215	9.0%	1.45 [0.91, 2.33]
Wilcock et al.,2000b	194	218	165	215	8.4%	2.45 [1.44, 4.16]
Wilkinson and Murray et al.,2001a	49	88	38	87	7.7%	1.62 [0.89, 2.94]
Wilkinson and Murray et al.,2001b	33	56	38	87	6.9%	1.85 [0.94, 3.65]
Wilkinson and Murray et al.,2001c	38	54	38	87	6.5%	3.06 [1.49, 6.30]
**Subtotal (95% CI)**		**2198**		**2134**	**92.4%**	**1.94 [1.47, 2.55]**
Total events	1771		1495			
Heterogeneity: Tau² = 0.15; Chi² = 33.43, df = 10 (P = 0.0002); I² = 70%						
Test for overall effect: Z = 4.69 (P < 0.00001)						
**5.1.2 The pool results of any adverse effects for all subgroups in severe group**						
Burns et al.,2009	183	207	177	200	7.6%	0.99 [0.54, 1.82]
**Subtotal (95% CI)**		**207**		**200**	**7.6%**	**0.99 [0.54, 1.82]**
Total events	183		177			
Heterogeneity: Not applicable						
Test for overall effect: Z = 0.03 (P = 0.98)						
**Total (95% CI)**		**2405**		**2334**	**100.0%**	**1.84 [1.41, 2.41]**
Total events	1954		1672			
Heterogeneity: Tau² = 0.15; Chi² = 36.47, df = 11 (P = 0.0001); I² = 70%						
Test for overall effect: Z = 4.48 (P < 0.00001)						
Test for subgroup differences: Chi² = 3.87, df = 1 (P = 0.05), I² = 74.2%						

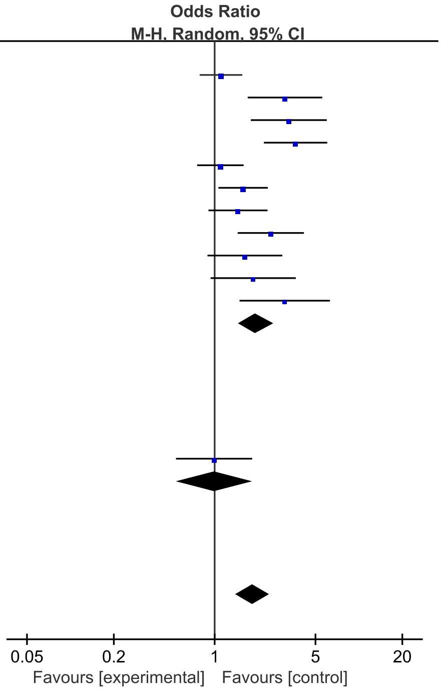

B

Study or Subgroup	Galantamine Events	Galantamine Total	Control Events	Control Total	Weight	Odds Ratio M-H, Random, 95% CI
**6.1.1 The pool results of any reason caused dropouts in mild to moderate group**						
Brodaty et al.,2005	75	326	54	320	10.3%	1.47 [1.00, 2.17]
Raskind et al.,2000a	68	212	41	213	9.5%	1.98 [1.27, 3.10]
Raskind et al.,2000b	89	211	41	213	9.6%	3.06 [1.98, 4.74]
Rockwood et al.,2001	86	261	12	125	7.1%	4.63 [2.42, 8.85]
Tariot et al.,2000a	60	279	46	286	9.8%	1.43 [0.93, 2.19]
Tariot et al.,2000b	61	273	46	286	9.8%	1.50 [0.98, 2.30]
Wilcock et al.,2000a	44	220	29	215	8.7%	1.60 [0.96, 2.68]
Wilcock et al.,2000b	55	218	29	215	8.9%	2.16 [1.32, 3.56]
Wilkinson and Murray et al.,2001a	25	88	14	87	6.2%	2.07 [0.99, 4.32]
Wilkinson and Murray et al.,2001b	14	56	14	87	5.4%	1.74 [0.76, 4.00]
Wilkinson and Murray et al.,2001c	26	54	14	87	5.8%	4.84 [2.21, 10.59]
**Subtotal (95% CI)**		**2198**		**2134**	**91.1%**	**2.08 [1.64, 2.63]**
Total events	603		340			
Heterogeneity: Tau² = 0.08; Chi² = 22.44, df = 10 (P = 0.01); I² = 55%						
Test for overall effect: Z = 6.06 (P < 0.00001)						
**6.1.2 The pool results of any reason caused dropouts in severe group**						
Burns et al.,2009	39	207	39	200	8.9%	0.96 [0.58, 1.57]
**Subtotal (95% CI)**		**207**		**200**	**8.9%**	**0.96 [0.58, 1.57]**
Total events	39		39			
Heterogeneity: Not applicable						
Test for overall effect: Z = 0.17 (P = 0.87)						
**Total (95% CI)**		**2405**		**2334**	**100.0%**	**1.95 [1.52, 2.50]**
Total events	642		379			
Heterogeneity: Tau² = 0.12; Chi² = 29.99, df = 11 (P = 0.002); I² = 63%						
Test for overall effect: Z = 5.28 (P < 0.00001)						
Test for subgroup differences: Chi² = 7.66, df = 1 (P = 0.006), I² = 86.9%						

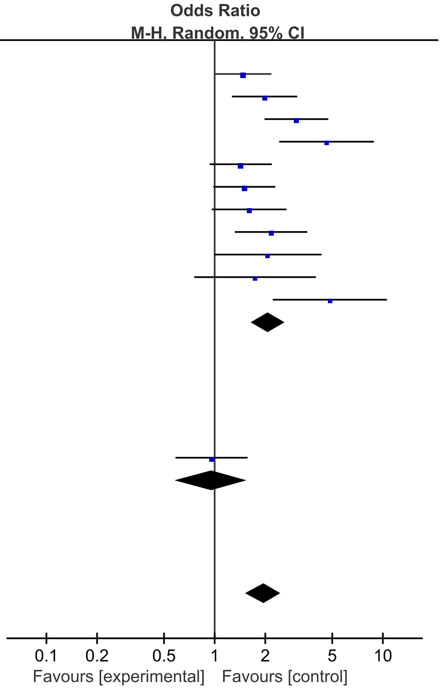

Figure S3. Safety and tolerability outcomes comparison on any adverse effects (A) and any reason caused dropouts (B) in the galantamine group versus the placebo group.

Study or Subgroup	Galantamine Events	Galantamine Total	Control Events	Control Total	Weight	Odds Ratio M-H, Random, 95% CI
**7.1.1 The pool results of adverse effects caused dropouts in mild to moderate group**						
Brodaty et al.,2005	24	326	15	320	8.6%	1.62 [0.83, 3.14]
Raskind et al.,2000a	49	212	16	213	9.0%	3.70 [2.03, 6.75]
Raskind et al.,2000b	67	211	16	213	9.1%	5.73 [3.19, 10.30]
Rockwood et al.,2001	66	261	5	125	7.1%	8.12 [3.18, 20.74]
Tariot et al.,2000a	19	279	20	286	8.7%	0.97 [0.51, 1.86]
Tariot et al.,2000b	27	273	20	286	9.0%	1.46 [0.80, 2.67]
Wilcock et al.,2000a	31	220	19	215	8.9%	1.69 [0.92, 3.10]
Wilcock et al.,2000b	48	218	19	215	9.1%	2.91 [1.65, 5.15]
Wilkinson and Murray et al.,2001a	19	88	8	87	7.3%	2.72 [1.12, 6.60]
Wilkinson and Murray et al.,2001b	10	56	8	87	6.7%	2.15 [0.79, 5.83]
Wilkinson and Murray et al.,2001c	24	54	8	87	7.2%	7.90 [3.20, 19.51]
**Subtotal (95% CI)**		**2198**		**2134**	**90.7%**	**2.73 [1.83, 4.09]**
Total events	384		154			
Heterogeneity: Tau² = 0.33; Chi² = 36.55, df = 10 (P < 0.0001); I² = 73%						
Test for overall effect: Z = 4.89 (P < 0.00001)						
**7.1.2 The pool results of adverse effects caused dropouts in severe group**						
Burns et al.,2009	30	207	31	200	9.3%	0.92 [0.54, 1.59]
**Subtotal (95% CI)**		**207**		**200**	**9.3%**	**0.92 [0.54, 1.59]**
Total events	30		31			
Heterogeneity: Not applicable						
Test for overall effect: Z = 0.28 (P = 0.78)						
**Total (95% CI)**		**2405**		**2334**	**100.0%**	**2.48 [1.64, 3.75]**
Total events	414		185			
Heterogeneity: Tau² = 0.40; Chi² = 48.76, df = 11 (P < 0.00001); I² = 77%						
Test for overall effect: Z = 4.30 (P < 0.0001)						
Test for subgroup differences: Chi² = 9.84, df = 1 (P = 0.002), I² = 89.8%						

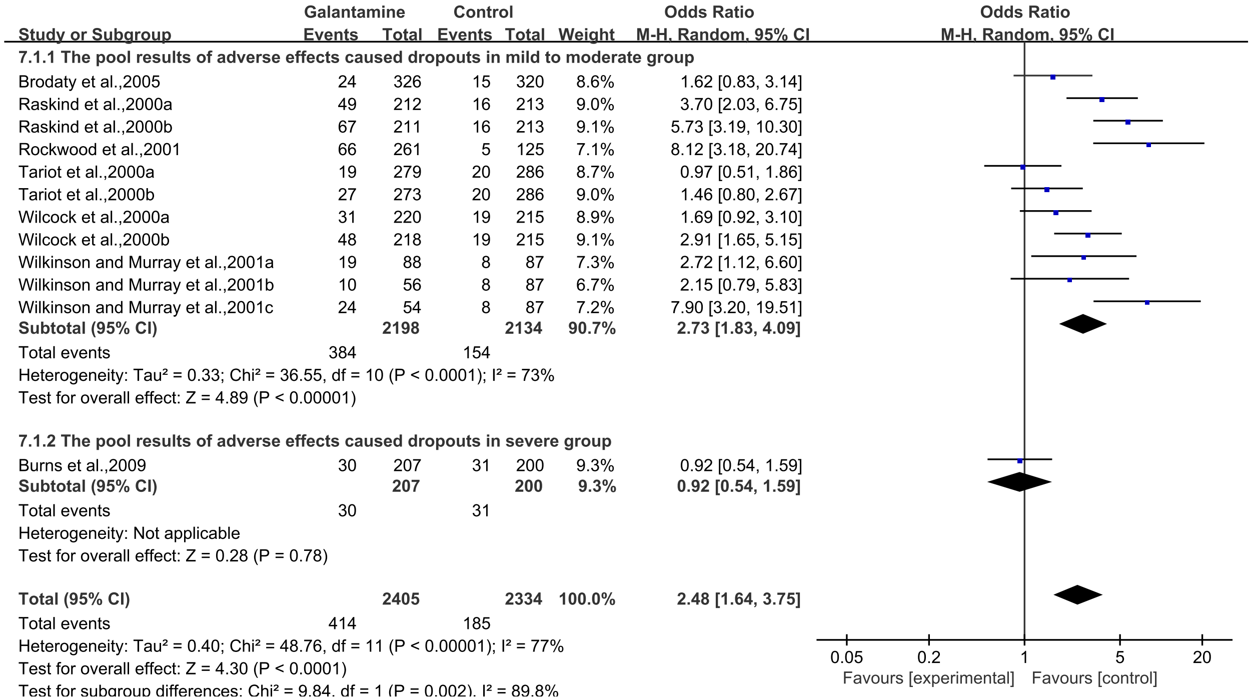

Figure S4: Safety and tolerability outcomes comparison on adverse effects caused dropouts in the galantamine group versus the placebo group.

A

Study or Subgroup	Rivastigmine Events	Rivastigmine Total	Control Events	Control Total	Weight	Odds Ratio M-H, Random, 95% CI
**6.1.1 The pool results of any reason caused dropouts in mild to moderate group**						
Feldman et al.,2007	24	227	20	222	27.8%	1.19 [0.64, 2.23]
Forette et al.,1999	9	23	1	19	5.6%	11.57 [1.31, 102.45]
Rosler et al.,1999	79	243	31	239	33.0%	3.23 [2.03, 5.14]
Winblad et al.,2007	63	297	36	302	33.6%	1.99 [1.27, 3.11]
**Subtotal (95% CI)**		**790**		**782**	**100.0%**	**2.24 [1.29, 3.88]**
Total events	175		88			
Heterogeneity: Tau² = 0.18; Chi² = 8.77, df = 3 (P = 0.03); I² = 66%						
Test for overall effect: Z = 2.86 (P = 0.004)						

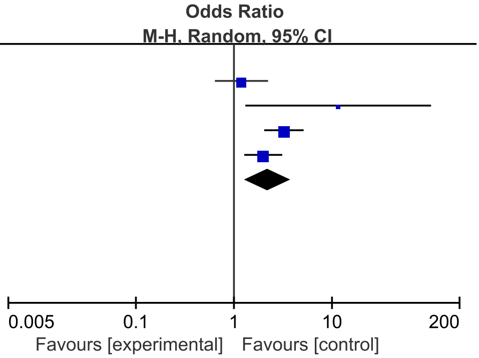

B

Study or Subgroup	Rivastigmine Events	Rivastigmine Total	Control Events	Control Total	Weight	Odds Ratio M-H, Random, 95% CI
**7.1.1 The pool results of adverse effects caused dropouts in mild to moderate group**						
Feldman et al.,2007	24	227	20	222	30.4%	1.19 [0.64, 2.23]
Forette et al.,1999	9	23	1	19	9.0%	11.57 [1.31, 102.45]
Rosler et al.,1999	55	243	16	239	31.1%	4.08 [2.26, 7.35]
Winblad et al.,2007	24	297	15	302	29.5%	1.68 [0.86, 3.27]
**Subtotal (95% CI)**		**790**		**782**	**100.0%**	**2.38 [1.12, 5.02]**
Total events	112		52			
Heterogeneity: Tau² = 0.38; Chi² = 10.81, df = 3 (P = 0.01); I² = 72%						
Test for overall effect: Z = 2.27 (P = 0.02)						

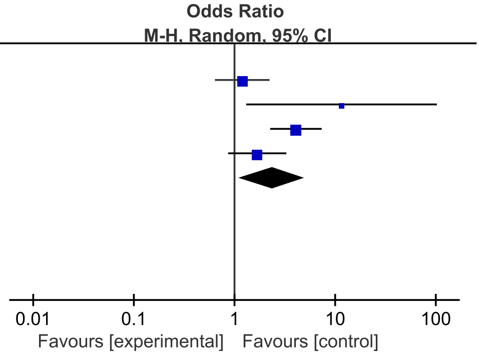

Figure S5. Safety and tolerability outcomes comparison on any reason caused dropouts (a) and adverse effects caused dropouts (b) in the rivastigmine group versus the placebo group.

A

Study or Subgroup	Memantine Events	Memantine Total	Control Events	Control Total	Weight	Risk Ratio M-H, Fixed, 95% CI
**5.1.1 The pool results of any adverse effects for all subgroups in mild to moderate group**						
Peskind et al.,2006	120	201	111	202	12.7%	1.09 [0.92, 1.29]
Porsteinssonet al.,2008	142	217	176	216	20.2%	0.80 [0.72, 0.90]
**Subtotal (95% CI)**		**418**		**418**	**32.9%**	**0.91 [0.83, 1.01]**
Total events	262		287			
Heterogeneity: Chi² = 8.78, df = 1 (P = 0.003); I² = 89%						
Test for overall effect: Z = 1.84 (P = 0.07)						
**5.1.2 The pool results of any adverse effects for all subgroups in moderate to severe group**						
Grossberg et al.,2013	214	341	214	335	24.7%	0.98 [0.88, 1.10]
Herrmann et al.,2013	138	182	136	187	15.4%	1.04 [0.92, 1.18]
Reisberg et al.,2003	106	126	109	126	12.5%	0.97 [0.88, 1.08]
van Dyck et al.,2007	131	178	125	172	14.6%	1.01 [0.89, 1.15]
**Subtotal (95% CI)**		**827**		**820**	**67.1%**	**1.00 [0.94, 1.06]**
Total events	589		584			
Heterogeneity: Chi² = 0.88, df = 3 (P = 0.83); I² = 0%						
Test for overall effect: Z = 0.03 (P = 0.98)						
**Total (95% CI)**		**1245**		**1238**	**100.0%**	**0.97 [0.92, 1.02]**
Total events	851		871			
Heterogeneity: Chi² = 13.86, df = 5 (P = 0.02); I² = 64%						
Test for overall effect: Z = 1.09 (P = 0.28)						
Test for subgroup differences: Chi² = 2.50, df = 1 (P = 0.11), I² = 60.1%						

Risk Ratio
M-H, Fixed, 95% CI

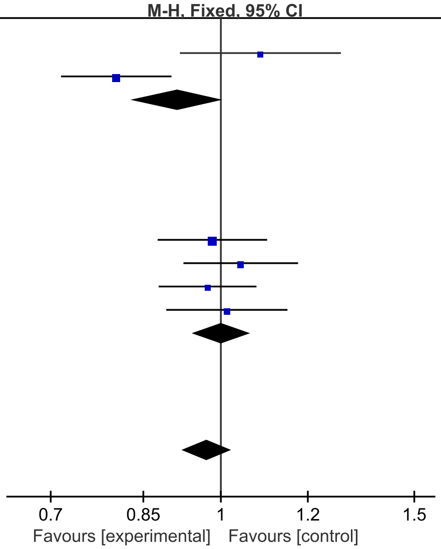

B

Study or Subgroup	Memantine Events	Memantine Total	Control Events	Control Total	Weight	Odds Ratio M-H, Fixed, 95% CI
**6.1.1 The pool results of any reason caused dropouts in mild to moderate group**						
Bakchine et al.,2008	47	318	14	152	6.0%	1.71 [0.91, 3.21]
Peskind et al.,2006	36	201	35	202	10.7%	1.04 [0.62, 1.74]
Porsteinssonet al.,2008	23	217	25	216	8.4%	0.91 [0.50, 1.65]
**Subtotal (95% CI)**		**736**		**570**	**25.1%**	**1.16 [0.83, 1.60]**
Total events	106		74			
Heterogeneity: Chi² = 2.27, df = 2 (P = 0.32); I² = 12%						
Test for overall effect: Z = 0.87 (P = 0.38)						
**6.1.2 The pool results of any reason caused dropouts in moderate to severe group**						
Fox et al.,2012	19	72	15	77	4.0%	1.48 [0.69, 3.20]
Grossberg et al.,2013	69	341	63	335	18.9%	1.10 [0.75, 1.60]
Herrmann et al.,2013	31	182	32	187	9.8%	0.99 [0.58, 1.71]
Reisberg et al.,2003	29	126	42	126	12.1%	0.60 [0.34, 1.04]
Tariot et al.,2004	30	203	51	201	16.3%	0.51 [0.31, 0.84]
van Dyck et al.,2007	44	178	46	172	13.2%	0.90 [0.56, 1.45]
Wang et al.,2013	2	13	2	13	0.6%	1.00 [0.12, 8.42]
**Subtotal (95% CI)**		**1115**		**1111**	**74.9%**	**0.86 [0.70, 1.05]**
Total events	224		251			
Heterogeneity: Chi² = 9.61, df = 6 (P = 0.14); I² = 38%						
Test for overall effect: Z = 1.46 (P = 0.15)						
**Total (95% CI)**		**1851**		**1681**	**100.0%**	**0.93 [0.79, 1.11]**
Total events	330		325			
Heterogeneity: Chi² = 13.91, df = 9 (P = 0.13); I² = 35%						
Test for overall effect: Z = 0.77 (P = 0.44)						
Test for subgroup differences: Chi² = 2.28, df = 1 (P = 0.13), I² = 56.1%						

Odds Ratio
M-H, Fixed, 95% CI

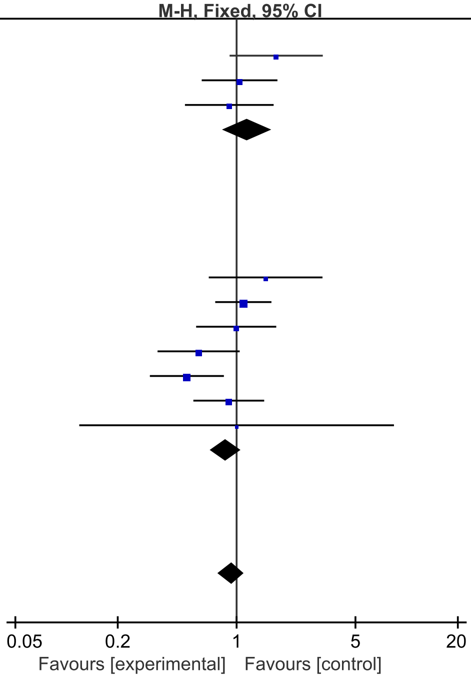

Figure S6. Safety and tolerability outcomes comparison on any adverse effects (A) and any reason caused dropouts (B) in the memantine group versus the placebo group.

Study or Subgroup	Memantine Events	Memantine Total	Control Events	Control Total	Weight	Odds Ratio M-H, Fixed, 95% CI
**7.1.1 The pool results of adverse effects caused dropouts in mild to moderate group**						
Bakchine et al.,2008	28	318	6	152	6.2%	2.35 [0.95, 5.80]
Peskind et al.,2006	19	201	10	202	7.6%	2.00 [0.91, 4.43]
Porsteinssonet al.,2008	13	217	17	216	13.5%	0.75 [0.35, 1.58]
**Subtotal (95% CI)**		**736**		**570**	**27.3%**	**1.46 [0.93, 2.29]**
Total events	60		33			
Heterogeneity: Chi² = 4.78, df = 2 (P = 0.09); I² = 58%						
Test for overall effect: Z = 1.66 (P = 0.10)						
**7.1.2 The pool results of adverse effects caused dropouts in moderate to severe group**						
Fox et al.,2012	3	77	4	72	3.3%	0.69 [0.15, 3.19]
Grossberg et al.,2013	34	342	21	335	16.0%	1.65 [0.94, 2.91]
Herrmann et al.,2013	15	182	10	187	7.6%	1.59 [0.69, 3.64]
Reisberg et al.,2003	22	126	13	126	9.0%	1.84 [0.88, 3.84]
Tariot et al.,2004	15	203	25	201	19.5%	0.56 [0.29, 1.10]
van Dyck et al.,2007	22	178	23	172	17.2%	0.91 [0.49, 1.71]
**Subtotal (95% CI)**		**1108**		**1093**	**72.7%**	**1.16 [0.87, 1.54]**
Total events	111		96			
Heterogeneity: Chi² = 9.03, df = 5 (P = 0.11); I² = 45%						
Test for overall effect: Z = 0.99 (P = 0.32)						
**Total (95% CI)**		**1844**		**1663**	**100.0%**	**1.24 [0.97, 1.58]**
Total events	171		129			
Heterogeneity: Chi² = 14.34, df = 8 (P = 0.07); I² = 44%						
Test for overall effect: Z = 1.74 (P = 0.08)						
Test for subgroup differences: Chi² = 0.74, df = 1 (P = 0.39), I² = 0%						

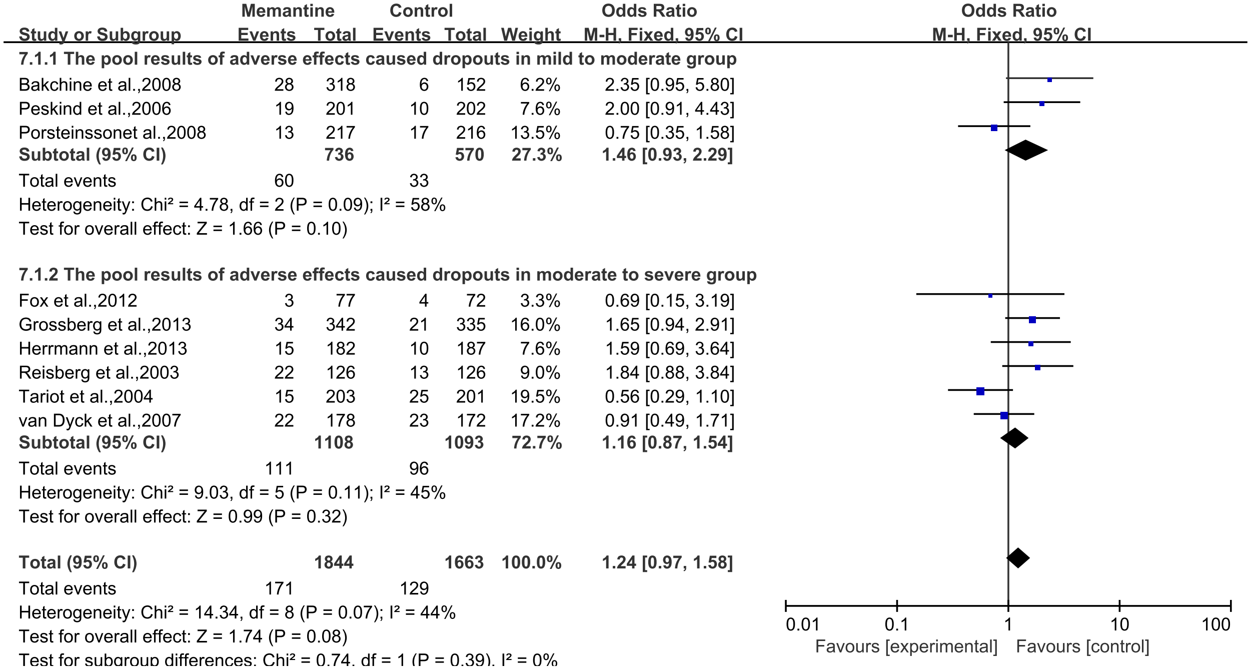

Figure S7: Safety and tolerability outcomes comparison on adverse effects caused dropouts in the memantine group versus the placebo group.
