# Supplementary material for: Meta-Analysis of Randomized Controlled Trials on the Efficacy and Safety of Donepezil, Galantamine, Rivastigmine, and Memantine for the Treatment of Alzheimer’s Disease
Source: Front Neurosci. 2019 May 15;13:472. doi: 10.3389/fnins.2019.00472 (PMC6529534; doi:10.3389/fnins.2019.00472)
Supplement: Supplementary file 2 [file Data_Sheet_2.PDF]

Supplementary Table 1. The characteristics of donepezil, galantamine, rivastigmine and memantine.

Medicine	Molecular Formula	Dosing range	Mechanism of action	Neurotransmitter system	Pharmacokinetics
Donepezil	$C_{24}H_{29}NO_3.HCl$	5-10 mg/dose, 1 dose/day	Reversibly binding to AChE	Ach	Metabolized by CYP3A4 and CYP2D6.
Galantamine	C17H21NO3	4-12 mg/dose, 2 doses/day	AchE inhibitor	Ach	Metabolized by CYP2D6
Rivastigmine	$C_{14}H_{22}N_2O_2$	1.5-6 mg/dose, 2 doses/day	Reversibly binding to ChE	Ach	Metabolized by its target ChE enzymes and is excreted through the urine.
Memantine	C12H21N	5-10 mg/dose, 1-2 doses/day	NMDA receptor antagonist	NMDA	Memantine is predominantly renally eliminated

Supplementary Table 2. The characteristics of ADAS-cog, ADCS-ADL, NPI and CIBIC+.

**Tests**	**Clinical application**	**Processing time**	**Score**	**Score interpretation**
ADAS-cog	Evaluate memory, language, and praxis	30 mins	0-70	Score is positively associated with AD
ADCS-ADL	Evaluate self care and daily function	5 mins	20-80	Score is positively associated with AD
NPI	Evaluate 12 neuropsychiatric disturbances	30 mins	0-144	higher scores indicating a greater neuropsychiatric disturbance
CIBIC+	Evaluate general, mental/cognitive state, behavior, and activities of daily living	5 mins	1-7	1 (marked improvement), 2 (moderate improvement), 3 (minimal improvement), 4 (no change), 5 (minimal worsening), 6 (moderate worsening), and 7 (marked worsening).

Supplementary Table 3. Baseline characteristics of the studies included in the meta-analysis, by study galantamine drug.

Study	Country	Dose (number of patient)	Gender (%men)	Age years (SD)	Disease severity	Type of Drug dosing	Duration (weeks)	Baseline MMSE (SD)	Outcomes				Dropout rate (%)	Number of adverse events caused dropout	Number of any adverse events
									Cognition	Function	Behavior	Global			
Tariot et al., 2000	USA	Placebo (286)	38	77.1 ± 8.5	Mild to moderate	Flexible	20	17.7 ± 3.4	ADAS-cog	ADCS-ADL	NPI	CIBIC+	16.1	20	206
a		16 mg daily (279)	35.4	76.3 ± 8.4				17.8 ± 3.3					21.5	19	206
b		24 mg daily (273)	33	77.7 ± 0.4				17.7 ± 3.3					22.3	27	219
Wilcock et al., 2000	UK	Placebo (215)	38.6	72.7 ± 7.6	Mild to moderate	Flexible	26	19.3 ± 3.5	ADAS-cog	-	-	CIBIC+	13.5	19	165
a		24 mg daily (220)	36.8	71.9 ± 8.3				19.5 ± 3.4					20.0	31	182
b		32 mg daily (218)	36.7	72.1 ± 8.6				19.0 ± 3.8					25.2	48	194
Raskind et al., 2000	USA	Placebo (213)	38.5	75.3 ± 8.8	Mild to moderate	Flexible	26	19.2 ± 4.4	ADAS-cog	-	-	CIBIC+	19.2	16	168
		24 mg daily (212)	34.4	75.9 ±7.3				19.5 ± 4.4					32.1	49	195
		32 mg daily (211)	41.2	75.0 ± 8.7				19.1 ± 4.4					42.2	67	195
Rockwood et al., 2001	Canada	Placebo (125)	46.4	74.6 ± 7.6	Mild to moderate	Flexible	12	19.6 ± 3.6	ADAS-cog	-	NPI	CIBIC+	9.6	5	79
		24-32 mg daily (261)	43.3	75.2 ± 7.3				19.7 ± 3.9					33.0	66	225
Wilkinson and Murray et al., 2001	UK	Placebo (87)	41.4	74.2 ± 8.4	Mild to moderate	Fixed	12	18.7 ± 2.8	ADAS-cog	-	-	-	16.1	8	38
a		18 mg daily (88)	44.3	72.7 ± 8.4				18.8 ± 2.8					28.4	19	49
b		24 mg daily (56)	41.1	72.9 ± 8.2				18.2 ± 3.0					25.0	10	33
c		36 mg daily (54)	42.6	75.4 ± 7.3				18.8 ± 3.7					48.1	24	38
Brodaty et al., 2005	Australia	Placebo (320)	36	76.3 ± 8.03	Mild to moderate	Fixed	28	18.1 ± 4.1	ADAS-cog	ADCS-ADL	NPI	CIBIC+	16.9	15	224
		24 mg daily (326)	36	76.5 ± 7.77				17.8 ± 4.1					23.0	24	235
Burns et al., 2009	UK	Placebo (200)	19	83.5 ± 5.8	Severe	Fixed	24	9.1 ± 2.4	-	ADCS-ADL	-	-	19.5	31	177
		24 mg daily (207)	19	83.7 ± 5.7				8.8 ± 2.4					18.8	30	183

ADAS-cog, Alzheimer's Disease (AD) Assessment Scale, cognitive subscale (possible range 0–70); ADCS-ADL, AD Cooperative Study Activities of Daily Living Inventory; ADCS-ADLsev, Alzheimer's Disease Cooperative Study Activities of Daily Living Inventory modified for severe dementia; CIBIC+, Clinicians' Interview-Based Impression of Change with Caregiver's Input (possible range 1–7); MMSE Mini-Mental State Examination, NPI Neuropsychiatric Inventory, a, b, c different doses of drug, - Not reported.

Supplementary Table 4. Baseline characteristics of the studies included in the meta-analysis, by study rivastigmine drug.

Study	Country	Dose (number of patient)	Gender (%men)	Age years (SD)	Disease severity	Type of Drug dosing	Duration (weeks)	Baseline MMSE (SD)	Outcomes				Dropout rate (%)	Number of adverse events caused dropout	Number of any adverse events
									Cognition	Function	Behavior	Global			
Rosler et al., 1999	Germany	Placebo (239)	41	72	Mild to moderate	Flexible	26	10–26	ADAS-cog	-	-	CIBIC+	13.0	16	-
		6-12mg daily (243)	-	-				-					32.5	55	-
Forette et al., 1999	France	Placebo (19)	-	72.5 ± 4.8	Mild to moderate	-	18	19.2	ADAS-cog	-	-		-	1	-
		10 mg daily (23)	-	69.5 ± 9.9				19.6					-	9	-
Feldman et al., 2007	UK	Placebo (222)	40	71.7 ± 8.7	Mild to moderate	Fixed	26	18.7 ± 4.6	ADAS-cog	-	-	CIBIC+	9.0	20	-
		12 mg daily (227)	40	71.4 ± 7.9				18.3 ± 4.5					10.6	24	-
Winblad et al., 2007	Japan	Placebo (302)	33.4	73.9 ± 7.3	Mild to moderate	Fixed	24	16.4 ± 3.0	ADAS-cog	ADCS-ADL	NPI	-	11.9	15	-
		12 mg daily (297)	34.4	72.8 ± 8.2				16.4 ± 3.1					21.2	24	-
		10 mg daily (60)	40.0	70.5 ± 8.31				18.1 ± 4.1					31.7	4	-

ADAS-cog, Alzheimer's Disease (AD) Assessment Scale, cognitive subscale (possible range 0–70); ADCS-ADL, AD Cooperative Study Activities of Daily Living Inventory; ADCS-ADLsev, Alzheimer's Disease Cooperative Study Activities of Daily Living Inventory modified for severe dementia; CIBIC+, Clinicians' Interview-Based Impression of Change with Caregiver's Input (possible range 1–7); MMSE Mini-Mental State Examination, NPI Neuropsychiatric Inventory, a, b, c different doses of drug, - Not reported.

Supplementary Table 5. Baseline characteristics of the studies included in the meta-analysis, by study memantine drug.

Study	Country	Dose (number of patient)	Gender (%men)	Age years (SD)	Disease severity	Type of Drug dosing	Duration (weeks)	Baseline MMSE (SD)	Outcomes				Dropout rate (%)	Number of adverse events caused dropout	Number of any adverse events
									Cognition	Function	Behavior	Global			
Reisberg et al., 2003	USA	Placebo (126)	34.5	75.8 ± 7.28	Moderate to severe	fixed	28	8.1 ± 3.6	-	ADCS-ADL$_{sev}$	NPI	-	33.3	13	109
		20 mg daily (126)	27.8	75.5 ± 8.16				7.8 ± 3.76					23.0	22	106
Tariot et al., 2004	UK	Placebo (201)	33	75.5 ± 8.73	Moderate to severe	fixed	24	10.2 ± 2.98	-	ADCS-ADL$_{19}$	NPI	CIBIC+	25.4	25	-
		20 mg daily (202)	37	75.5 ± 8.45				9.9 ± 3.13					14.8	15	-
Peskind et al., 2006	USA	Placebo (202)	42.6	77.0 ± 8.2	Mild to moderate	fixed	24	17.2 ± 3.4	ADAS-cog	ADCS-ADL$_{23}$	NPI	-	17.3	10	15
		20 mg daily (201)	39.8	78.0 ± 7.3				17.4 ± 3.7					17.9	19	15
van Dyck et al., 2007	USA	Placebo (172)	29.7	78.3 ± 7.6	Moderate to severe	fixed	24	10.3 ± 3.1	-	ADCS-ADL$_{19}$	NPI	-	26.2	23	125
		20mg daily (178)	27.5	78.1 ± 8.2				10.0 ± 2.8					24.7	22	131
Porsteinsson et al.,2008	USA	Placebo (216)	49.5	76.0 ± 8.43	Mild to moderate	fixed	24	17.0 ± 3.63	ADAS-cog	ADCS-ADL$_{23}$	NPI	-	11.6	17	15
		10 mg daily (217)	46.1	74.0 ± 7.64				16.7 ± 3.68					10.6	13	22
Bakchine et al., 2008	France	Placebo (152)	40	73.3 ± 6.9	Mild to moderate	fixed	24	18.9 ± 3.2	ADAS-cog	ADCS-ADL$_{23}$	NPI	CIBIC+	9.2	28	80
		20 mg daily (318)	35	74.0 ± 7.4				18.6 ± 3.3					1.3	6	178
Fox et al., 2012	UK	Placebo (77)	24.7	84.4 ± 6.6	Moderate to severe	fixed	12	7.3 ± 6.4	-	-	NPI	-	19.5	4	-
		10 mg daily (72)	27.8	84.9 ± 6.7				7.3 ± 6.2					26.4	3	-
Wang et al., 2013	China	Placebo (11)	36	64.7 ± 11.5	Moderate to severe	Fixed	24	10.1 ± 6.1	ADAS-cog	-	-	-	2.0	-	-
		10 mg daily (11)	36	65.7 ± 12.5				14.1 ± 4.6					2.0	-	-
Grossberg et al., 2013	USA	Placebo (335)	27.5	76.8 ± 7.8	Moderate to severe	fixed	24	10.6 ± 2.9	-	ADCS-ADL$_{19}$	NPI	-	18.8	21	214
		28mg daily ER (341)	28.4	76.2 ± 8.4				10.9 ± 2.9					20.2	34	214
Herrmann et al., 2013	Canada	Placebo (187)	41.2	75.1 ± 6.9	Moderate to severe	fixed	24	11.8 ± 2.9	-	-	NPI	-	17.1	10	136
		20 mg daily (182)	42.3	74.7 ± 7.9				11.9 ± 3.1					17.0	15	138

ADAS-cog, Alzheimer's Disease (AD) Assessment Scale, cognitive subscale (possible range 0–70); ADCS-ADL, AD Cooperative Study Activities of Daily Living Inventory; ADCS-ADLsev, Alzheimer's Disease Cooperative Study Activities of Daily Living Inventory modified for severe dementia; CIBIC+, Clinicians' Interview-Based Impression of Change with Caregiver's Input (possible range 1–7); MMSE Mini-Mental State Examination, NPI Neuropsychiatric Inventory, a, b, c different doses of drug, - Not reported.

Supplementary Table 6. The comparison of the published meta-analysis with the present study.

Study	Donepezil				Galantamine				Rivastigmine				Memantine			
	Cognition	Function	Behavior	Global	Cognition	Function	Behavior	Global	Cognition	Function	Behavior	Global	Cognition	Function	Behavior	Global
Bond *et.al*, 2012	+ (12 weeks)	+ (24-28 weeks)	-	+	+	+	+ (13weeks) - (21-26 weeks)	+ (26 weeks)	+	+	+/-	+ (26 weeks)	+	+	-	+
Di Santo *et.al,* 2013	+	+	o	o	+	+	o	o	+	+	o	o	+	+	o	o
Loveman *et.al,* 2006	+	+/-	-	+	+	+	+/-	+/-	+	+/-	-	+	+/-	+	+/-	+
Raina *et.al*, 2008	+	o	o	+	+	o	o	+	+	o	o	+	+	o	o	+
the present study	+	+	-	+	+	+	+	+	+	-	-	+	+	+	-	-

"+", statistically significance; "-", no statistically significance; "+/-", controversial result; "o", not reported.
